# Dysregulation of STAT3 signaling is associated with endplate-oriented herniations of the intervertebral disc in *Adgrg6* mutant mice

**DOI:** 10.1371/journal.pgen.1008096

**Published:** 2019-10-25

**Authors:** Zhaoyang Liu, Garrett W. D. Easson, Jingjing Zhao, Nadja Makki, Nadav Ahituv, Matthew J. Hilton, Simon Y. Tang, Ryan S. Gray

**Affiliations:** 1 Department of Nutritional Sciences, University of Texas at Austin, Austin, Texas, United States of America; 2 Department of Pediatrics, Dell Pediatric Research Institute, University of Texas at Austin Dell Medical School, Austin, Texas, United States of America; 3 Department of Orthopedics, Washington University School of Medicine, Saint Louis, Missouri, United States of America; 4 Department of Bioengineering and Therapeutic Sciences and Institute for Human Genetics, University of California San Francisco, San Francisco, California, United States of America; 5 Department of Anatomy and Cell Biology, University of Florida, College of Medicine, Gainesville, Florida, United States of America; 6 Department of Orthopedic Surgery and Cell Biology, Duke University School of Medicine, Durham, North Carolina, United States of America; University of Southern California, UNITED STATES

## Abstract

Degenerative changes of the intervertebral disc (IVD) are a leading cause of disability affecting humans worldwide and has been attributed primarily to trauma and the accumulation of pathology during aging. While genetic defects have also been associated with disc degeneration, the precise mechanisms driving the initiation and progression of disease have remained elusive due to a paucity of genetic animal models. Here, we discuss a novel conditional mouse genetic model of endplate-oriented disc herniations in adult mice. Using conditional mouse genetics, we show increased mechanical stiffness and reveal dysregulation of typical gene expression profiles of the IVD in *adhesion G-protein coupled receptor G6* (*Adgrg6*) mutant mice prior to the onset of endplate-oriented disc herniations in adult mice. We observed increased STAT3 activation prior to IVD defects and go on to demonstrate that treatment of *Adgrg6* conditional mutant mice with a small molecule inhibitor of STAT3 activation ameliorates endplate-oriented herniations. These findings establish ADGRG6 and STAT3 as novel regulators of IVD endplate and growth plate integrity in the mouse, and implicate ADGRG6/STAT3 signaling as promising therapeutic targets for endplate-oriented disc degeneration.

## Author summary

Back pain is a leading cause of disability in humans worldwide and one of the most common culprits of these issues are the consequence of degenerative changes of the intervertebral disc. Here, we demonstrate that conditional loss of the *Adgrg6* gene in cartilaginous tissues of the spine results in endplate-oriented disc herniations and degenerative changes of the intervertebral disc in mice. We further establish that these obvious degenerative changes of the disc are preceded by substantial alterations in normal gene expression profiles, including upregulation of pro-inflammatory STAT3 signaling, and increased mechanical stiffness of the intervertebral disc. Increased STAT3 activation is a signal observed in other models of degenerative musculoskeletal tissues. As such, we tested whether systemic treatment with a small-molecule STAT3 inhibitor would protect against the formation of endplate-oriented disc herniations in conditional *Adgrg6* mutant mice, and report a significant positive improvement of histopathology in our treatment group. Taken together, we demonstrate a novel conditional model of endplate-oriented disc herniation in mouse. We establish ADGRG6 and STAT3 as novel regulators of endplate integrity of the intervertebral disc in mouse and suggest that modulation of ADGRG6/STAT3 signaling could provide robust disease-modifying targets for endplate-oriented disc degeneration in humans.

## Introduction

Spine disorders are one of the most common health issues affecting human populations worldwide, causing a tremendous socio-economic burden. The progression of spine disorders such as low back pain, disc herniation, endplate fracture, and scoliosis are associated with degenerative changes of the intervertebral disc (IVD) [1–4]. Therefore, elucidation of the pathways and signaling important for maintaining spine stability and the development and homeostasis of the IVD tissues is critical for the diagnosis, prevention, and treatment of degenerative spine disorders.

**Fig 1:**
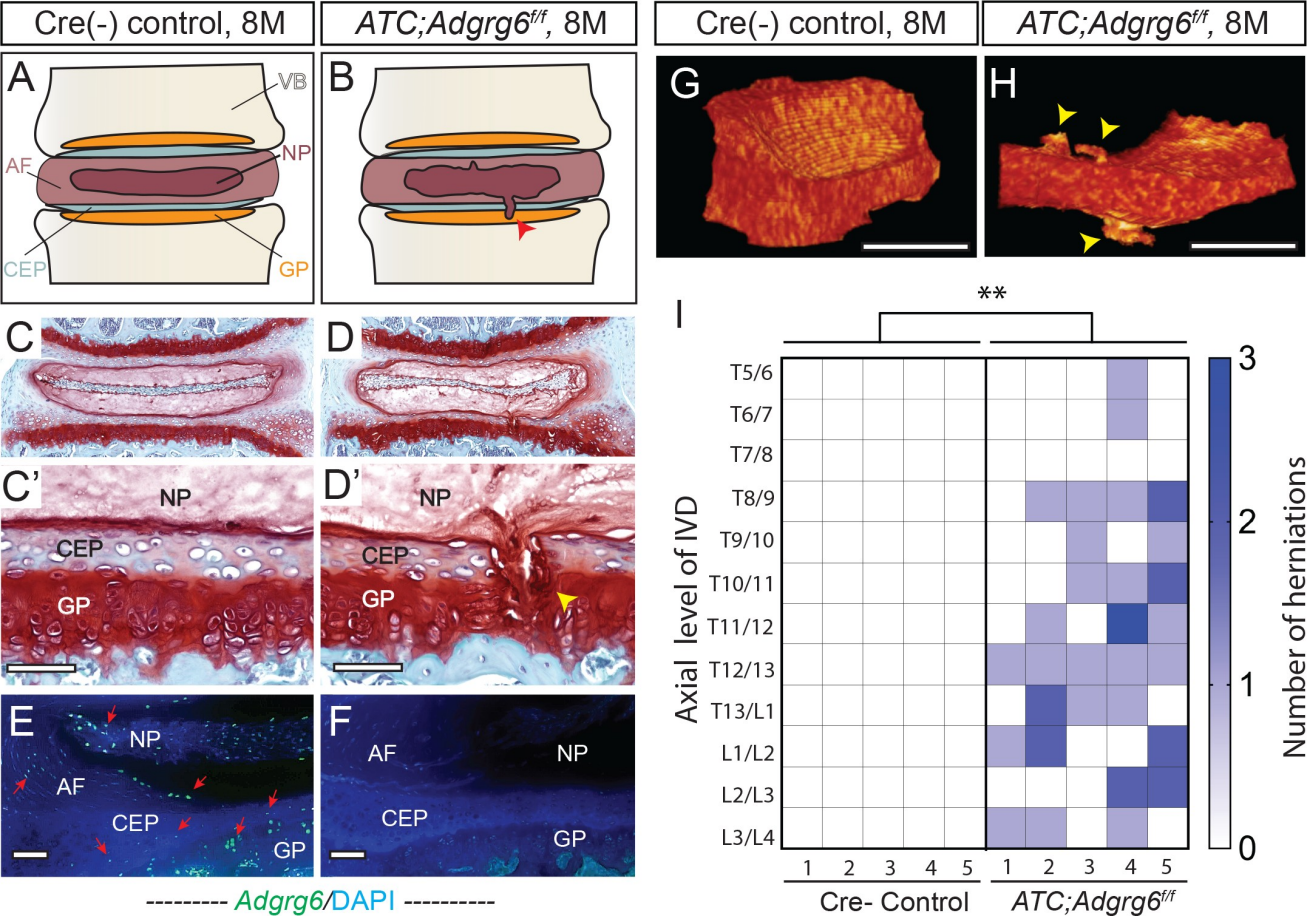
Adult *ATC;Adgrg6*^*f/f*^ mutant mice display endplate-oriented herniations of the IVD. (A and B) Schematic of endplate-oriented herniations (B, red arrowhead) observed in *ATC;Adgrg6*^*f/f*^ mutant mice (B), in contrast to a typical wild-type IVD (A) at 8 months of age. (C-D’) Representative midline-sectioned 8-month-old mouse IVDs stained with Safranin-O/Fast green (SO/FG) (induced from P1-P20, *n* = 4 for each group). (E, F) *Adgrg6* riboprobe FISH (green fluorescence) at 8 months (induced from P1-P20, *n* = 3 for each group). (G, H) Representative reconstructions of contrast-enhanced μCT of Cre (-) control (G) and *ATC;Adgrg6*^*f/f*^ mutant (H) IVDs at 8 months of age (induced from E0.5-P20, *n* = 5 for each group). Endplate-oriented herniations are observed in SO/FG stained sections (D', yellow arrowhead) and by contrast-enhanced μCT (H, yellow arrowheads). (I) Heat map of contrast-enhanced μCT data from five Cre (-) control and five *ATC;Adgrg6*^*f/f*^ mutant mouse spines, plotting the axial level of the IVD (left axis) and the number of herniations (right axis) observed in each mouse. (**p≤0.01, two-tailed Student's *t* Test.) Scale bars: 50μm in (C’, D’), 100μm in (E, F), and 500μm in (G, H). *AF- annulus fibrosis*, *CEP- cartilaginous endplate*, *GP- growth plate*, *NP- nucleus pulposus*, *and VB- vertebral body*.

The IVD is a fibrocartilaginous joint that connects two adjacent vertebrae and provides structural stability, flexibility, and cushions axial loading of the spinal column [5]. The disc is composed of a proteoglycan-rich nucleus pulposus, surrounded by a multi-lamellar annulus fibrosus, and situated between the cartilaginous endplates, which provide nutritional flux to the IVD (Fig 1A). Hallmarks of disc degeneration in humans include loss of disc height, reduced proteoglycan staining, and accumulation of markers of fibrosis within the disc. At the same time, the cartilaginous endplate (CEP) may show signs of degeneration and calcification, which further compromises nutrient availability to the inner disc layers [6]. More severe forms of disc degeneration can also result in the herniation of the nucleus pulposus (i) laterally through the annulus fibrosis layer; or (ii) through the cartilaginous endplate into the vertebral body (endplate-oriented). Genetic susceptibility to disc degeneration has been shown to play a major role in disc degeneration [7], with the majority of these findings implicating extracellular matrix components of the disc, matrix metalloproteases, or pro-inflammatory cytokines [8]. Together these data suggest that dysregulation of anabolic and catabolic factors as well as inflammatory signaling may underlie many forms of disc degeneration in humans. However, the molecular regulators and initiating factors for these events remain to be defined.

Here, we show that *Adgrg6* has a critical role in endplate-oriented herniations of the disc through regulation of STAT3 signaling. ADGRG6 (also called GPR126) is a member of the adhesion G-protein coupled receptor (aGPCR) family of proteins, all of which are thought to have a canonical intercellular signaling function via G-protein coupled signaling, as well as a potential for cell-cell or cell-matrix signaling via the extracellular N-terminal fragment [9]. In zebrafish, *adgrg6/gpr126* is critical for the development of cartilaginous tissues of the semicircular canal via regulation extracellular matrix (ECM) gene expression [10], suggesting a role for ADGRG6 in the regulation of cartilaginous tissues. Conditional loss of *Adgrg6* in multipotent osteochondral progenitors -giving rise to bone, cartilage, and some connective tissues- of the spine generated postnatal-onset scoliosis, ribcage deformity, and increased incidence of midline clefts in the endplates and annulus fibrosus [11]. Since the development of scoliosis is often linked with IVD deformity [12], we sought to investigate the role of *Adgrg6* specifically in cartilaginous tissues of the IVD during embryonic and postnatal development.

To define the role of *Adgrg6* we combined conditional mouse genetics, genomic approaches, mechanical assessment of intervertebral disc, and cell biological approaches in chondrogenic cell culture. Together, these studies reveal that ADGRG6 has a conserved role for the maintenance of normal gene expression profiles and regulation of STAT3 signaling in cartilaginous cells. We demonstrate that loss of *Adgrg6* leads to increased expression of collagens associated with fibrosis and alteration of the normal biomechanical properties of the IVD, prior to the onset of endplate-oriented herniations. Finally, we demonstrate loss of *Adgrg6* leads to increased, ectopic STAT3 activation in the IVD and that blockade of STAT3 activation can decrease the incidence of endplate-oriented herniations in *Adgrg6* conditional mutant mice. Taken together, our work establishes *Adgrg6* as a novel regulator of IVD endplate and growth plate integrity in the mouse and suggests that modulation of ADGRG6/STAT3 signaling could provide robust disease-modifying targets for endplate-oriented disc degeneration, in humans.

## Results

### Loss of ADGRG6 in intervertebral discs leads to endplate-oriented herniations in adult mice

We demonstrate that conditional removal of ADGRG6 function in the intervertebral disc (IVD) results in endplate-oriented herniations in adult mice (Fig 1B, 1D and 1D’). We found that *Adgrg6* is highly expressed in the growth plate using immunohistochemistry-based *in situ* hybridization (S1A Fig), but we failed to detect expression in the cortical or trabecular bone in vertebrae of adult mice under these conditions. Using a more sensitive, fluorescent *in situ* hybridization (FISH) detection method we were also able to detect *Adgrg6* expression in cells of the cartilaginous endplate (CEP), annulus fibrosus, and nucleus pulposus (Figs 1E and S1C). To determine the role of ADGRG6 function specifically in these committed chondrogenic lineages of the spine, we utilize an Aggrecan enhancer-driven, Tetracycline-inducible Cre (*ATC*) transgenic mouse strain [13] (*ATC;Adgrg6*^*f/f*^). Using this inducible Cre-deleter strain we established two experimental groups to address the temporal requirement of ADGRG6 function by induction during embryonic development (from E0.5-P20, prior to IVD specification) or during perinatal development (from P1-P20, after IVD specification) (S1 Table).

Using either induction strategy, we consistently observed endplate-oriented disc herniations [14] in adult mutant mice at 8 months of age (Figs 1 and S2B and S2B’ and S4D). *ATC;Adgrg6*^*f/f*^ mutant mice induced during perinatal development (P1-P20) displayed prolapse of the nucleus pulposus material into the growth plate (Figs 1B, 1D and 1D’ and S4D), as did mutant mice recombined during embryonic development (E0.5-P20) (S2B and S2B’ Fig). These data suggest that the formation of these endplate-oriented disc herniations is largely attributed to loss of ADGRG6 function during postnatal development. Interestingly, histological analysis of *ATC;Adgrg6*^*f/f*^ mutant IVDs, regardless of timing of induction, did not reveal observable changes in disc height, alterations of overall sulfated-proteoglycan abundance (Safranin-O staining) (Figs 1D and S2 and S4), or any obvious defects of the annulus fibrosis or nucleus pulposus tissues when visualized at the midline of the intervertebral disc at 8 months (Figs 1D and S2C and S4D’). In contrast, endplate-oriented herniations were observed at various spatial levels of the IVD including at the midline (Fig 1D and 1D) and at more lateral sections of the IVD (S2B, S2B’ and S4D Figs), suggesting a unique endplate-driven pathology for this model.

In this way, we found that traditional two-dimensional histological analysis limited our ability to capture the extent and distribution of these endplate-oriented herniations along the spine. To address this, we exploited contrast-enhanced micro-computed tomography (μCT) imaging which allows for a full three-dimensional analysis and segmented visualization of the IVD within the intact mouse spine [15]. Reconstruction and segmentation of the whole IVD in 8-month-old *ATC;Adgrg6*^*f/f*^ mutant (Fig 1H and S1 Movie) and Cre (-) control mice (Fig 1G and S2 Movie) (49 discs total; *n* = 5 mice/genotype) revealed multiple incidences of endplate-oriented disc herniations (yellow arrowheads, Fig 1H). Quantification of the contrast-enhanced μCT imaging indicated from 0–3 herniations present/IVD in *ATC;Adgrg*^*f/f*^ mutant mice, while Cre (-) littermate control mice were not observed to display these defects (Fig 1I). Spatial analysis of the intact spine demonstrated that these herniations occurred along the entire axis of the spine (Thoracic (T)5/6—Lumbar (L)3/4), without obvious hotspots. As was demonstrated by histology, we did not observe radial fissures or lateral prolapse of the disc in *ATC;Adgrg6*^*f/f*^ mutant mice using contrast-enhanced microCT imaging. This further supports IVD degeneration in this conditional mutant mouse is occurring by endplate-driven mechanisms [14].

**Fig 2:**
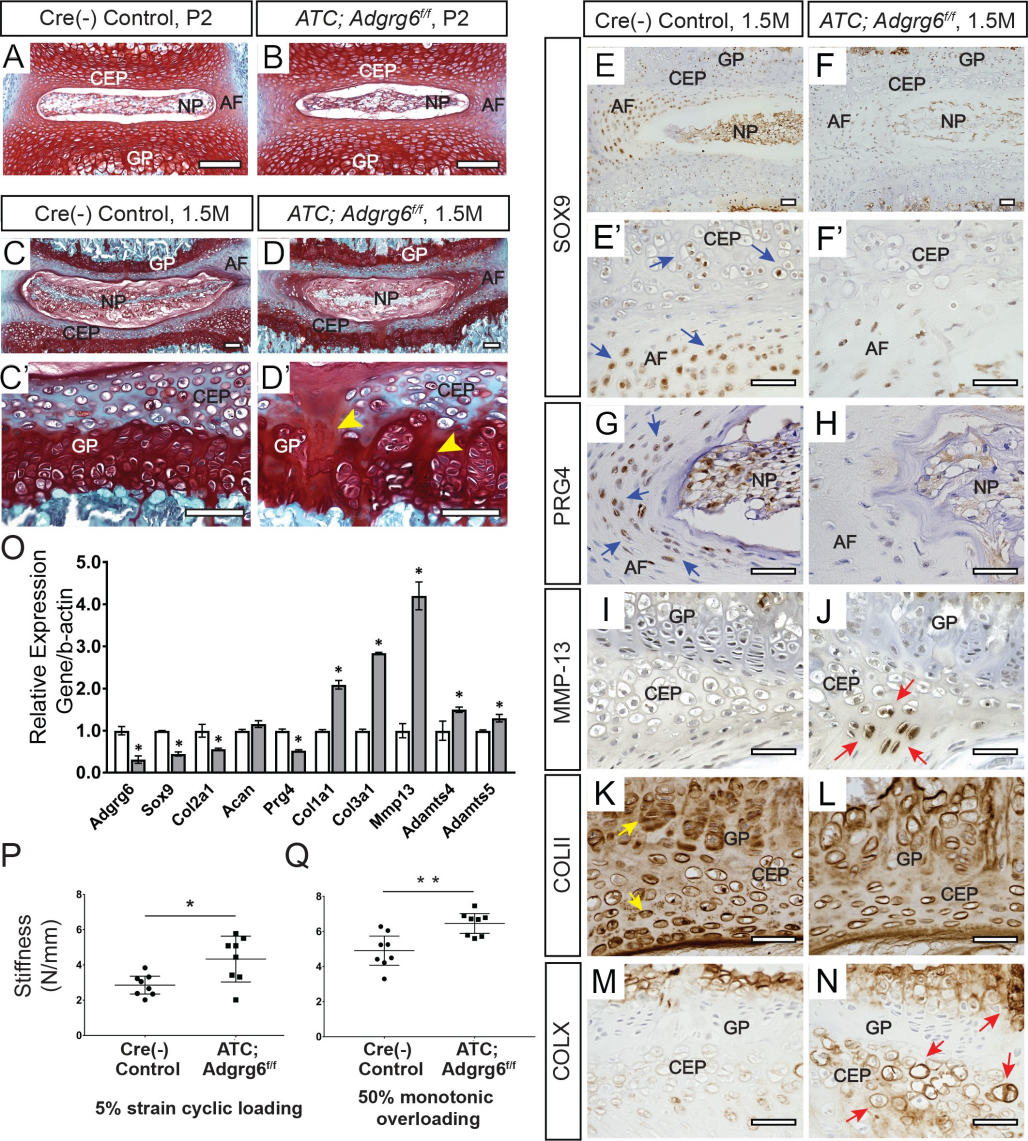
Young *ATC;Adgrg6*^*f/f*^ mutant mice display alterations in IVDs consistent with disc degeneration pathology. (A-D’) Representative IVD tissues stained Safranin-O/Fast green (SO/FG) (Induced from E0.5-P20, *n* = 3 for each group.) No overt structural defects were observed in *ATC;Adgrg6*^*f/f*^ mutant IVDs compared with controls at P2 at midline (A, B) or at 1.5 months of age (C-D’), except for the mild increase of acellular clefts in the CEP and GP at 1.5 months (yellow arrowheads, D’). Section in panel D is a little bit pass midline to show the obvious acellular clefts in CEP and GP. (E-O) IHC (E-N) and qPCR (O) analyses of common markers of degenerative disc in mice at 1.5 months (induced from E0.5-P20). *ATC;Adgrg6*^*f/f*^ mutant IVDs display reduced expression of markers of healthy disc: SOX9/*Sox9* (blue arrows, E’) and PRG4/*Prg4* (blue arrows, G), and mildly reduced COLII/*Col2a1* (yellow arrows, K). They also display increased expression of hypertrophic marker COLX/*Col10a1* (red arrows, N), extracellular matrix modifying enzymes (MMP-13/*Mmp13* (red arrows, J), *Adamts4*, and *Adamts5*), fibrosis markers (*Col1a1* and *Col3a1*). (E-N, *n* = 3 for each group. O, *n* = 3 biological replicates and representative result is shown. Bars represent mean ± SD. *p≤0.05, two-tailed Student's *t* Test.) (P, Q) Mechanical testing using 5% strain cyclic loading (stiffness mean w/95% CI, *p < 0.05) (P), and 50% monotonic overloading (stiffness mean w/95% CI, **p < 0.01) (Q), demonstrating increased stiffness in *ATC;Adgr6g*^*f/f*^ mutant lumbar IVDs (induced from E0.5-P20, *n* = 4 for each group, 4 IVDs were analyzed /mouse). Scale bars: 100μm in (A-D’); 50μm in (E-N). *AF- annulus fibrosis*, *CEP- cartilaginous endplate*, *GP- growth plate*, *and NP- nucleus pulposus*.

In previous studies we demonstrated a clear role for *Adgrg6* in the formation of late-onset scoliosis in mouse [11], however the cellular pathogenesis of this process remained unresolved. Interestingly, while ~80% of *Col2Cre;Adgrg6*^*f/f*^ mutant mice demonstrated late-onset scoliosis [11], we only observed late-onset scoliosis in ~12% of *ATC;Adgrg6*^*f/f*^ mutant mice (S1 Table). One possible explanation is the difference in targeted tissues between the two Cre-deleter strains. Analysis of recombination using β-galactosidase staining of *ATC;Rosa-LacZ* mice showed nearly complete recombination throughout the IVD and growth plate at weaning (P28) regardless of the timing of induction (S3C and S3D Fig). However, the outer most layers of the annulus fibrosus and the periosteum were not targeted using either strategy (S3A, S3C and S3D Fig). In contrast, the entire IVD as well as periosteum and trabecular bone in the vertebral body is completely recombined at P1 and P28 when crossed to the *Col2Cre* deleter strain (S3B and S3E Fig). Effective knockdown of *Adgrg6* in *ATC;Adgrg6*^*f/f*^ mutant mice (induced from E0.5-P20) was further confirmed using FISH analysis of *Adgrg6* expression at 8 months (Figs 1F and S1D and S1F) and by qPCR analysis of cDNA derived from IVDs extracted from 1.5 month-old mice (Fig 2O). *In situ* hybridization analysis showed that *Adgrg6* expression was not altered in tissues not recombined by the ATC-deleter strain, such as the periosteum (S1E and S1F Fig). Together these data suggest that the IVD is important for susceptibility of scoliosis, yet additional effectors of spine stability are involved. Importantly, irrespective of the incidence of scoliosis in the thoracic spine in young mice (~12%, observed around P20-P40), *ATC;Adgrg6*^*f/f*^ mutant mice consistently exhibited endplate-oriented herniations along the entire axis of the spine in adult mice (100%, *n* = 5) (Fig 1I). These data demonstrate that ADGRG6 has a unique role in the regulation of endplate-oriented herniations of the adult IVD, in addition to its role in scoliosis.

### Loss of ADGRG6 in the intervertebral discs leads to alterations of gene expression consistent with human disc degeneration pathology

We next sought to understand the molecular mechanism underlying the initiation of endplate-oriented herniations. To guide our analysis, we took cues from molecular changes reported in degenerative IVDs in human [16], which revealed several indicators of degenerative joint disease in *ATC;Adgrg6*^*f/f*^ mutant mice at 1.5 months, prior to overt histopathology (Fig 2D and 2D’, induced from E0.5-P20). Using immunohistochemistry (IHC) we observed a consistent reduction in the expression of the transcription factor SOX-9 (SOX9) (Figs 2F and 2F’ and S5B) and proteoglycan 4 (PRG4) (Figs 2H and S5D) in the inner layer of the annulus fibrosus and CEP in these mutant mice. In contrast, the expression of the hypertrophic chondrocyte marker type X collagen (COLX) was increased in the CEP and hypertrophic growth plate in *ATC;Adgrg6*^*f/f*^ mutant mice (Figs 2N and S5J). We also observed a mild reduction of type II collagen (COLII) staining in the pericellular matrix of chondrocytes in the CEP, and in the proliferative growth plate in mutant mice (Figs 2L and S5H). We observed mild increases of matrix metalloprotease-13 (MMP-13) expression in the CEP of *ATC;Adgrg6*^*f/f*^ mutant mice (Figs 2J and S5F). Analysis of *ATC;Adgrg6*^*f/f*^ mutant mice induced after birth (P1-P20) demonstrated similar alterations in protein expression in the IVD at 8 months (S4E–S4L Fig). Alterations of protein expression in the CEP, coupled with the consistency of endplate-oriented herniations in *ATC;Adgrg6*^*f/f*^ mutant mice regardless of embryonic or perinatal induction of recombination (Figs 1 and S2 and S4), strongly suggest a postnatal role for ADGRG6 function in the homeostasis of the CEP. These alterations in protein expression were supported by similar changes in gene expression as assayed by qPCR analysis of extracted IVDs (Fig 2O) which demonstrate reduced expression of *Col2a1*, *Sox9* and *Prg4* in *ATC;Adgrg*^*f/f*^ mutant mice at 1.5 months. Moreover, the expression of several catabolic mediators of degeneration including *Mmp13*, *Adamts4*, and *Adamts5*, as well as markers of fibrosis, *Col1a1* and *Col3a1*, were increased. Together, these alterations of typical gene expression profiles in the IVD are consistent with expression profiles reported for degenerative joint diseases, including degenerative discs [17] and osteoarthritis of articulating joints [18, 19] in humans.

As demonstrated previously, ADGRG6 is not critical for early pattering or the overall morphology of the IVD [11], however it does regulate perinatal and homeostatic processes of the CEP and growth plate (Fig 1D and 1D’ and 2D and 2D’). To underscore this, we show that embryonic recombination of the *ATC;Adgrg6*^*f/f*^ mutant mice, which removes *Adgrg6* function specifically in cartilaginous tissues, does not affect the specification or overall pattern of the IVD nor lead to obvious alternations of the Safranin-O staining of the disc at P2 (Fig 2B). However, mild morphological differences in nucleus pulposus and delays in midline fusion of the CEP are occasionally observed in embryonically-induced *ATC;Adgrg6*^*f/f*^ mutant mice at P2, consistent with our previous findings using the *Col2Cre*-deleter strain [11], which may reflect a mild developmental delay in these mutant mice. By 1.5 months, we begin to observe a mild increase in acellular clefts in the CEP and growth plate in *ATC;Adgrg6*^*f/f*^ mutant mice (7.2±3.4 clefts/IVD; *n* = 4; *p* = 0.03) (Fig 2D'), compared to littermate controls (2.5±0.4 clefts/IVD; *n* = 4) (Fig 2C'). This is consistent with our observations of *ATC;Adgrg6*^*f/f*^ mutant mice, recombined perinatally, which also demonstrate acellular clefts in growth plate at 4-months (40%; *n* = 5) (yellow arrows, S4B Fig). Finally, TUNEL staining demonstrated a mild increase in cell death in *ATC;Adgrg6*^*f/f*^ mutant mice within the annulus fibrosus, nucleus pulposus, and CEP compartments of the IVD, less so within vertebral growth plate at 1.5 months (S6B and S6C Fig). Taken together these data support that while ADGRG6 function is important in cartilaginous tissues of the spine it is not a critical factor for overall development of the IVD. In contrast, it seems to have an increasingly important role within the CEP and growth plate for perinatal development and adult homeostasis of these tissues.

### Mechanical properties are altered prior to the onset of obvious histopathology in the intervertebral discs of *Adgrg6* mutant mice

During the progression of disc degeneration and osteoarthritis-related joint remodeling in humans, increased catabolic factors, inflammatory signaling, coupled with alterations of normal extracellular matrix composition can have deleterious effects on the mechanical properties on these tissues [20]. In order to assess alterations of mechanical properties of *ATC;Adgrg6*^*f/f*^ mutant IVDs, we isolated individual lumbar discs (L1/2 and L4/5) for dynamic mechanical testing (16 discs total; *n* = 4 mice/genotype) from 1.5-month-old mutant and control mice (induced from E0.5-P20). Using micro-indentation we demonstrated a consistent increase in the stiffness (Newton (N)/mm) of *ATC;Adgrg6*^*f/f*^ mutant IVDs under 5% strain cyclic loading (Fig 2P; *p* = 0.0114; mean w/95% CI) and under 50% monotonic overloading (Fig 2Q; *p* = 0.0026; mean w/95% CI). Stiffening of the IVD is commonly observed with early-onset degenerative changes, which compromises the damage resistance of the tissue [21]. We analyzed proteoglycan quantification in these IVDs by dimethylmethylene blue (DMMB) assay and found no significant alterations comparing mutant and littermate control mice at 1.5 months. Similarly, we observed no alteration in total collagen content in *ATC;Adgrg6*^*f/f*^ mutant IVDs by hydroxyproline assay (measured as collagen/wet weight; *p* = 0.0561; one-tailed *t*-test). However, we observed increased expression collagens associated with fibrosis in 1.5-month-old mutant IVDs, correlated with a decrease in the expression of typical collagen genes observed in healthy cartilage (Fig 2O). We speculate that, alterations of typical extracellular matrix/collagen gene and protein expression, coupled with increased cell death contribute a constellation of factors leading to the decline in the normal mechanical properties of the IVD in *ATC;Adgrg6*^*f/f*^ mutant mice.

### Loss of *Adgrg6* in the intervertebral disc leads to alterations of collagen gene expression and alterations in ion transport components

**Fig 3:**
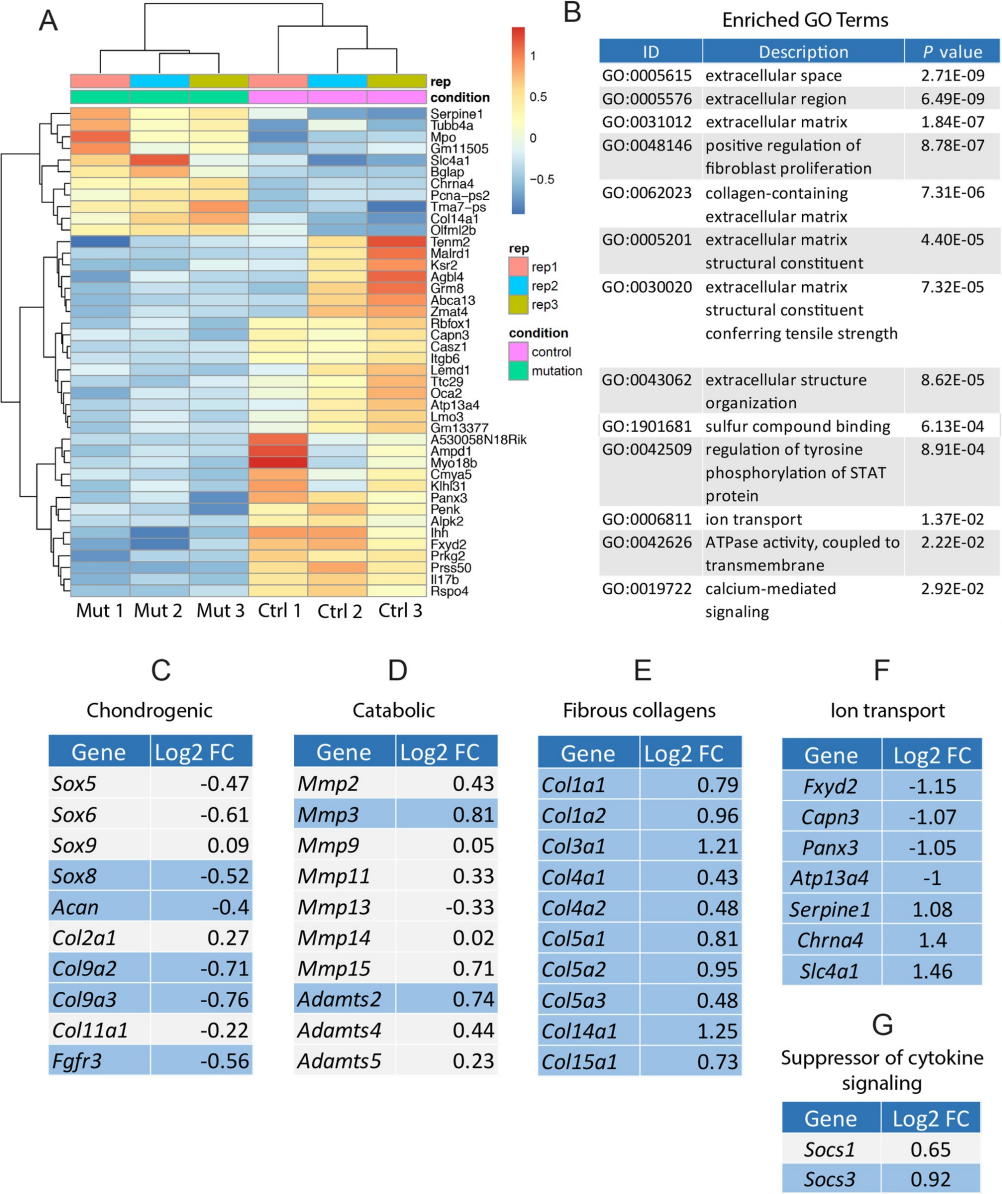
Young *Col2Cre;Adgrg6*^*f/f*^ mutant mice display fibrotic-like changes of gene expression and dysregulation of genes associated with ion transport in the IVD. (A) Heatmap of differentially expressed gene based on RNA-sequencing analysis of IVDs derived from both *Col2Cre;Adgrg6*^*f/f*^ mutant (Mut 1–3) and Cre (-) littermates (Ctrl 1–3) at P20. (B) Gene ontology (GO) analysis revealed a suite of differentially expressed genes important for extracellular matrix organization and ion transport. (C-G) RNA-sequencing analysis revealed mild alterations in some chondrogenic (C) and catabolic (D) gene expression, but significantly induced fibrotic gene expression (E) and dysregulation of genes involved in ion transport (F) in the *Col2Cre;Adgrg6*^*f/f*^ mutant IVDs at P20. Some genes encode members of the suppressor of cytokine signaling were also upregulated in the mutant IVDs (G). Differential expressed genes with p value < 0.05 were highlighted in blue. Log2FC: gene expression fold changes in Log2 scale.

To obtain additional, unbiased insights of the cellular and molecular changes in *Adgrg6* conditional mutant IVDs prior to overt histopathology, we applied transcriptomic analysis (RNA-seq) on whole IVDs extracted from mice at P20. To avoid the contamination of untargeted IVD tissue in the *ATC;Adgrg6*^*f/f*^ mutant mice (e.g. the outer most annulus fibrosus, S3C and S3D Fig), we choose to use *Col2Cre;Adgrg6*^*f/f*^ mutant mice for these studies as this experimental group completely recombines throughout the entire IVD during embryonic development (S3B and S3E Fig). At P20, *Col2Cre;Adgrg6*^*f/f*^ mutant mice display no overt histopathology at the midline in any IVD tissues or in the growth plate, akin to Cre(-) littermate control IVDs (S7A and S7B’ Fig). However, in 8-month-old *Col2Cre;Adgrg6*^*f/f*^ mutant mice we observe endplate-oriented herniations (S7D’ Fig), analogous to observations in *ATC;Adgrg6*^*f/f*^ mutant IVDs (Fig 1). Similarly, we do not observe overt defects of the annulus fibrosus and nucleus pulposus in *Col2Cre;Adgrg6*^*f/f*^ mutant IVDs when imaged at the midline of the IVD (S7E Fig). We generated three independent libraries for each genotype (using *Col2Cre;Adgrg6*^*f/f*^ mutants and Cre (-) littermate controls) from extracted IVD tissues (T8/9-L4/5), pooled from 2–3 individual mice at P20. We found 884 differential expressed genes with statistical significance (*p* value < 0.05) (S2 Table), and with a more stringent cut-off adjusted *p* value <0.05 and fold-change ≥2, we observed 42 differential expressed genes (Fig 3A). Enrichment of pathways and biological processes using gene ontology (GO) terms [22] demonstrated associations with extracellular matrix, positive regulation of fibroblast proliferation, extracellular matrix structural constituent conferring tensile strength, regulation of tyrosine phosphorylation of STAT protein, and ion transport (Fig 3B). Several of the significantly upregulated genes are associated with risk of lumbar disc degeneration and osteoarthritis assayed in humans or animal models, including *Aspn*, *Dkk-3*, and *Mmp3* [23–27] (S2 Table). We also observed alterations of chondrogenic and catabolic gene expression (Fig 3C and 3D). Surprisingly some of the genes altered in *ATC;Adgrg6*^*f/f*^ mutant mice at 1.5 months by qPCR analysis (Fig 2O), such as *Sox9*, *Col2a1* and *Mmp13*, were not similarly changed in slightly younger *Col2Cre;Adgrg6*^*f/f*^ mutant mice (P20) by RNA-Seq analysis. However, in both *ATC* and *Col2Cre* conditional *Adgrg6* mutant mouse models we observed consistent alteration of collagen expression, including *Col1a1*, *Col3a1* among others at P20 in *Col2Cre;Adgrg6*^*f/f*^ mutant mice (Fig 3E) and at 1.5 months in *ATC;Adgrg6*^*f/f*^ mutant mice (Fig 2O), resulting in a shift in collagen gene expression, including type II collagen, towards more fibrous collagen gene expression associated with degenerative IVD from both human and mouse models [28–31]. We suggest that increased expression of these collagens may drive increased stiffness of the IVD observed in *ATC;Adgrg6*^*f/f*^ mutant mice at 1.5 months (Fig 2P and 2Q) which in turn may contribute to increased susceptibility of endplate-oriented disc herniations in adult mutant mice.

In addition, we observed gene expression changes in several genes associated with ion transport in the IVD of *Col2Cre;Adgrg6*^*f/f*^ mutant mice at P20, including reduced expression of *Fxyd2*, *Capn3*, *Panx3*, and *Atp13a4*, as well as increased expression of *Serpine1*, *Chrna4*, and *Slc4a1* (Fig 3F). High osmotic pressure is a characteristic of the IVD [32] and ion channel activity plays a critical role in the regulation of osmotic changes [33, 34]. In agreement, recent analysis of the SM/J isotype mouse model of disc degeneration mice is associated with gene expression changes in ion transport systems [31]. Altogether, our transcriptomic analysis of the IVDs in *Col2Cre;Adgrg6*^*f/f*^ mutant mice demonstrated a robust dysregulation of several important pathways and components of the IVD homeostasis, including collagen gene expression, alteration of ion transport components, as well as increased expression of several established catabolic factors prior to the onset of histopathology and disc degeneration. These data suggest that ADGRG6 signaling is a critical regulator of postnatal homeostasis of the CEP and growth plate.

### ADGRG6 regulates STAT3 signaling in cartilaginous cells

RNA-Seq analyses also implicate pro-inflammatory signaling is involved in *Adgrg6* mutant IVDs. For example, pathways associated with inflammation and activation/phosphorylation of STAT proteins (GO: 0042509—regulation of tyrosine phosphorylation of STAT protein) were altered (Fig 3B) and the expression of *suppressor of cytokine signaling* (*Socs*) genes, *Socs3 and Socs1* are expressed 1.9 and 1.6 fold higher in *Col2Cre;Adgrg6*^*f/f*^ mutant mice respectively (Fig 3G). SOCS3 is known to directly regulate STAT1 (signal transducer and activator of transcription 1) and STAT3 activation [35]. SOCS3 also acts as a negative feedback effector inhibiting IL-6 production which acts to inhibit prolonged IL-6/STAT-3 signaling [36]. Finally, STAT3/IL-6 signaling has been previously implicated in IVD degeneration [37, 38]. For these reasons, we wanted to assay STAT1 and STAT3 activity in the IVD of *ATC;Adgrg6*^*f/f*^ mutant mice.

**Fig 4:**
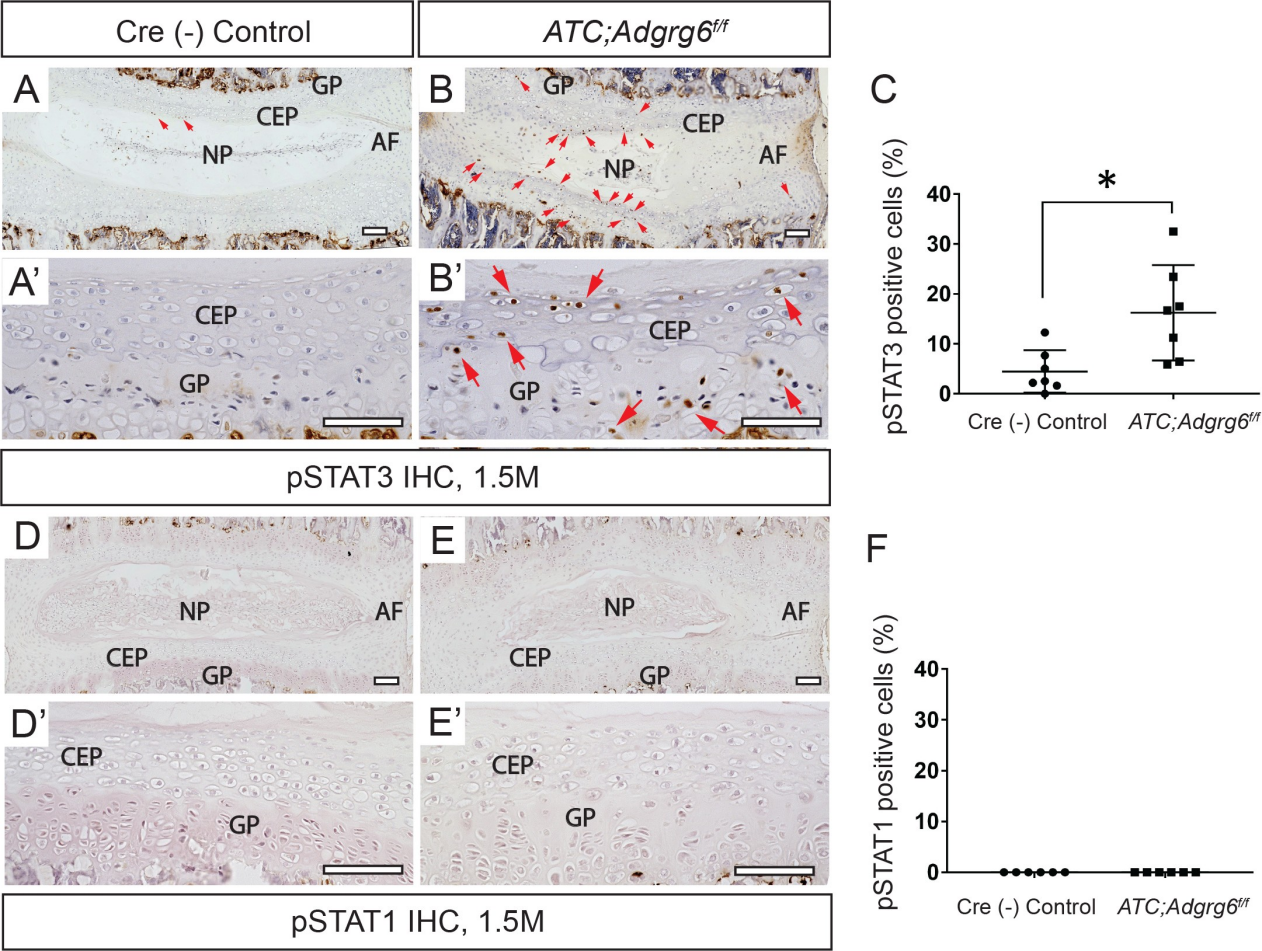
ADGRG6 regulates STAT3 signaling in IVDs. (A-B’) IHC analysis shows increased expression of pSTAT3 (red arrowheads, B, B’) in *ATC;Adgrg6*^*f/f*^ mutant mouse IVD at 1.5 months (induced from E0.5-P20, *n* = 3 for each group.) (D-E’) IHC analysis shows no signal of pSTAT1 in neither Cre(-) nor *ATC;Adgrg6*^*f/f*^ mutant mouse IVD at 1.5 months (induced from E0.5-P20, *n* = 3 for each group.) (C, F) Quantification of positive pSTAT3 (C) and pSTAT1 (F) cells in Cre(-) control or in *ATC;Adgrg*^*f/f*^ mutant mouse IVDs. (*n* = 3 mice for each group, at least two IVDs were scored for each mouse. Dots plot with mean ± SD. *p≤0.05, two-tailed Student's *t* Test.) Scale bars: 200μm in (A, B), and 50μm in (A’, B’). *CEP- cartilaginous endplate*, *GP- growth plate*, *and NP- nucleus pulposus*.

By IHC analysis, we observed a substantial increase in phosphorylated STAT3 (pSTAT3) signal, the active form of STAT3 protein, in the CEP and the growth plate of the *ATC;Adgrg6*^*f/f*^ mutant mice at 1.5 months (Fig 4B and 4B’), prior to overt histopathology of the disc. Quantification analysis revealed that 16.7% of cells in the mutant IVD are pSTAT3 positive (*n* = 3 mice; 2–3 IVDs/mouse; *n* = 526 cells total), in comparison to 5.2% of the control IVD (*n* = 3 mice; 2–3 IVDs/mouse; *n* = 417 cells total) (Fig 4C). Similar analysis using an antibody against pSTAT1 failed to detect any signal in either genotype (Fig 4D–4F). Together these data demonstrate that ADGRG6 regulates STAT3 activation in the CEP and growth plate of the IVD.

To better understand the origin of STAT3 activation we analyzed the distribution of macrophages, one of the key immune cells elicited by inflammatory response [39], by checking a well-established macrophage marker CD68 on IVD sections of the *ATC;Adgrg6*^*f/f*^ mutant IVD at 1.5 months. We did not observe any accumulation of macrophages at the CEP and growth plate (S8A and S8B Fig) in these mutant mice, demonstrating that the STAT3 activation is not due to peripheral macrophage infiltration into the IVD. We also assayed CD68 on the 8-month-old *ATC;Adgrg6*^*f/f*^ mutant IVD, but only observe minor signal at the site of herniation (S8D Fig), indicating that endplate-oriented herniations within the growth plate does not invoke a robust immune response.

**Fig 5:**
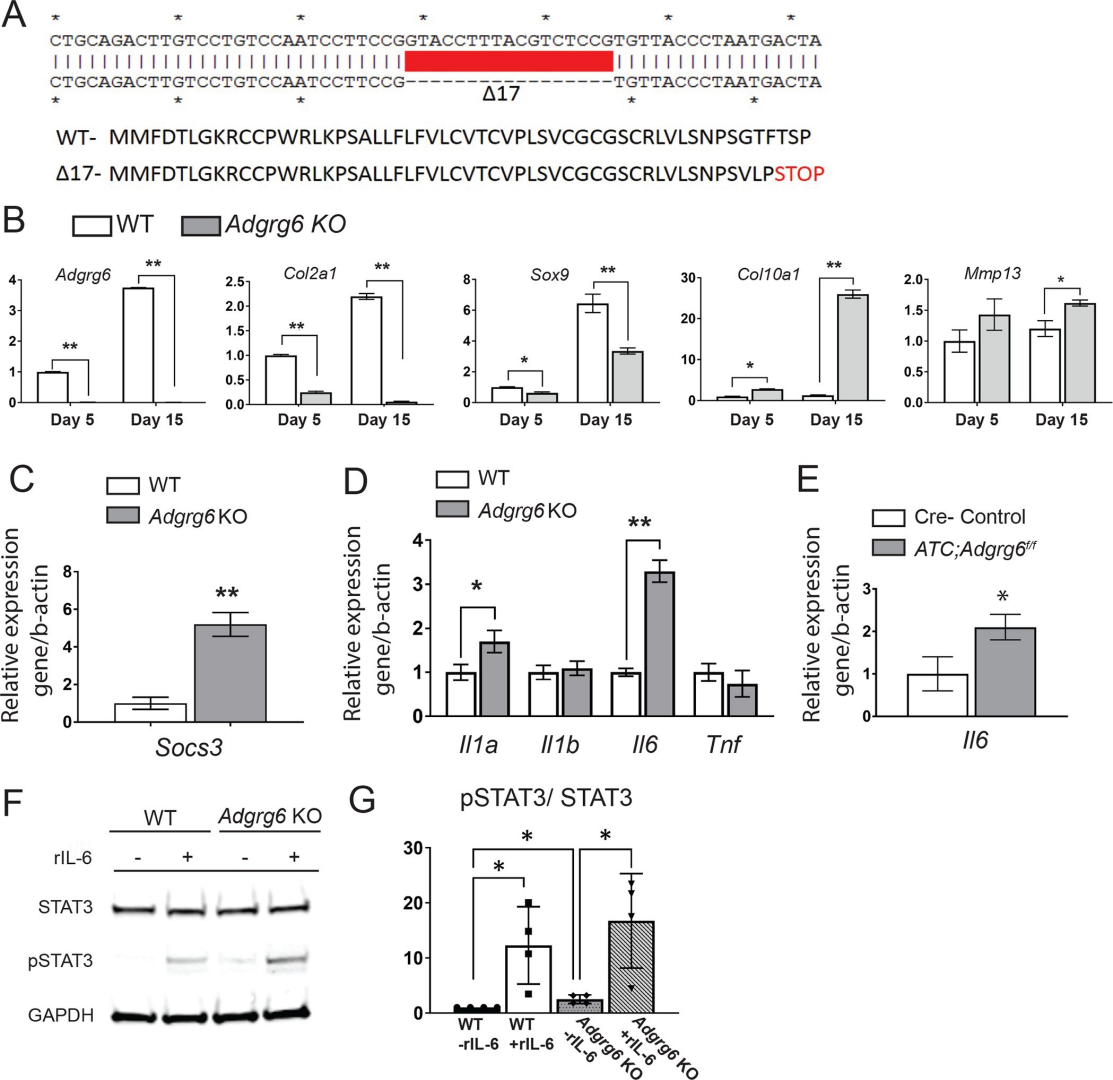
*Adgrg6* regulates gene expression profiles and STAT3 signaling in ATDC5 cell culture. (A) Schematic of a 17-bp deletion of *Adgrg6* from a stable single cell clone of ATDC5 cell line (*Adgrg6* KO). (B) qPCR analyses of gene expression in ATDC5 cells at 5 and 15 days of maturation demonstrates decreased expression of markers of healthy disc, *Col2a1*and *Sox9*, and increased expression of the hypertrophic marker, *Col10a1* and the extracellular matrix modifying enzyme, *Mmp13* in *Adgrg6* KO cells. (C) qPCR analyses revealed increased expression of *Socs3* in *Adrgr6* KO cells after 15 days of maturation. (D) qPCR analysis of *Il1a*, *Il1b*, *Il6* and *Tnf* in ATDC5 cells maturated for 15 days. (E) qPCR analysis of *Il6* expression in 1.5-month-old primary mouse IVDs (induced from E0.5-P20). (B-E, *n* = 3 biological replicates and representative result is shown. Bars represent mean ± SD. *p≤0.05, **p≤0.01, two-tailed Student's *t* Test). (F) Representative Western blot of wild type and *Adgrg6* KO ATDC5 cell lysates showing stimulation of pSTAT3 staining after treatment with recombinant IL-6 (rIL-6) protein in both cell lines, while *Adgrg6* KO cells show a mild constitutive stimulation of pSTAT3 without addition of rIL-6 (*n* = 4 biological replicates and representative result is shown). (G) Densitometry of the Western blot of wild type and *Adgrg6* KO ATDC5 cell with or without IL-6 (rIL-6) treatment. (Each dot represents one biological replicate. Bars plot with mean ± SD. *p≤0.05, two-tailed Student's *t* Test.)

In order to facilitate more mechanistic studies of ADGRG6 function in chondrogenic tissues, we utilized the ATDC5 mouse cell line which can be induced to form cartilage-like tissue *in vitro* [40]. After 15 days of maturation, the wild type ATDC5 cells robustly express chondrogenesis markers (*Col2a1* and *Sox9*), but not the hypertrophic chondrocyte marker (*Col10a1*) (Figs 5B and S9), which is analogous to cartilaginous cells in CEP and proliferative growth plate. We utilized lentivirus driven CRISPR-Cas9 technique in ATDC5 cells and engineered a stable INDEL mutant with a homozygous 17-bp deletion in exon 3 of *Adgrg6*, predicted to generate a frameshift mutation at amino acid Ser49 resulting in a truncated ADGRG6 protein (Adgrg6^p.Ser49+3fs^) (Fig 5A). The complete reduction of *Adgrg6* expression in our clonal *Adgrg6* mutant cell line (*Adgrg6* KO) suggested a null allele, likely due to non-sense mediated decay of the transcript (Fig 5B). During the course of chondrogenic maturation in unedited ATDC5 cells, *Adgrg6* expression increases with similar kinetics as other chondrogenic markers *Col2a1*, *Sox9* and *Acan* (S9 Fig). Consistent with our observations *in vivo* (Fig 2E–2N and 2O), we observed reduced expression of chondrogenic markers *Sox9* and *Col2a1*, and mild induction of the catabolic enzyme *Mmp13* in *Adgrg6* KO cells, demonstrating a common function of *Adgrg6* in maintaining gene expression profiles in chondrogenic lineages (Fig 5B). We also observed increased expression of hypertrophic marker *Col10a1* in the ATDC5 KO cells (Fig 5B), suggesting precocious maturation and inappropriate initiation of hypertrophy upon loss of ADGRG6 function. Indeed, similar precocious maturation phenotype was observed in *Sox9* haploinsufficiency mice [41], and we also found that the expression level of *Mef2c*, another key regulation of chondrocyte hypertrophy [13, 42], is upregulated in *Adgrg6* KO cells (5.2 fold compared with wild type control, p<0.05), which may also contribute to the induced *Col10a1* expression. Taken together, our data suggest that *Adgrg6* plays a critical role in regulation of normal gene expression profiles in chondrogenic cells. In addition, cleaved-Caspase 3, a key effector of apoptosis, was increased 2-fold in *Adgrg6* KO cells after 10-day maturation (S10 Fig), consistent with our observations of increased cell death in *ATC;Adgrg6*^*f/f*^ mutant mice IVDs (S6 Fig). Consistent with the RNA-seq analysis of *Col2Cre;Adgrg6*^*f/f*^ conditional mutant mice at P20 (Fig 3), we also observed a 5-fold increase in the expression of *Socs3* in *Adgrg6* KO cells (Fig 5C), while *Socs1* was not detectable in either unedited wild-type or *Adgrg6* KO ATDC5 cells. Taken together, the strong correlation between these *in vivo* and *in vitro* findings suggests a cell autonomous function for ADGRG6 for the regulation of typical gene expression profiles and STAT3 activation in cartilaginous cells.

Cytokines including interleukin-6 (IL-6), IL-1, and Tumor necrosis factor alpha (TNF), are known to induce STAT3 phosphorylation through receptor-associated Janus kinases. pSTAT3 can then translocate to the nucleus to regulate many cellular processes, such as cell growth and apoptosis [43]. To better understand the mechanism of ADGRG6-dependent regulation of STAT3 activation, we assayed a panel of known pro-inflammatory cytokines *Il1a*, *Il1b*, *Il6* and *Tnf* in *Adgrg6* KO cells maturated for 15 days, observing increased expression of both *Il1a* and *Il6* expression (Fig 5D). Importantly, increased expression of *Il6* was also observed in *ATC;Adgrg6*^*f/f*^ mutant IVDs at 1.5 months of age (Fig 5E).

IL-6 can not only stimulate the production of catabolic enzymes, but also can suppress the expression of anabolic genes, including *Sox9*, *Col2a1*, and *Acan* [44]. To determine whether increased *Il6* expression was coupled with activation of STAT3 upon loss of ADGRG6 function, we assayed the ability of ATDC5 cells to respond to recombinant IL-6 protein (rIL-6) during chondrogenic maturation *in vitro*. Western blot analysis demonstrated that rIL-6 effectively stimulates increased expression of pSTAT3 in both unedited wild type and *Adgrg6* KO cells (Fig 5F and 5G). Interestingly, we revealed a low level of increased pSTAT3 expression in *Adgrg6* KO cell lysates that were not stimulated by rIl-6 (Fig 5F and 5G). These *in vitro* results further demonstrate that ADGRG6 regulates STAT3 activation in cartilaginous cells in a cell autonomous manner through increased paracrine and/or autocrine mediated IL-6 signaling.

### STAT3 blockade protects against the formation of endplate-oriented herniations in *Adgrg6* mutant mice

To further define the role of STAT3 signaling on the pathogenesis of endplate-oriented disc degeneration, we next tested if systemic inhibition of STAT3 activation could ameliorate these defects *in vivo*. We choose to utilize the constitutive *Col2Cre;Adgrg6*^*f/f*^ conditional mutant mice for this experiment in order to limit stress on the mice and avoid exposure to Dox as potential confounding variable prior to long-term (16-week) treatment with the STAT3 inhibitor, Stattic.

**Fig 6:**
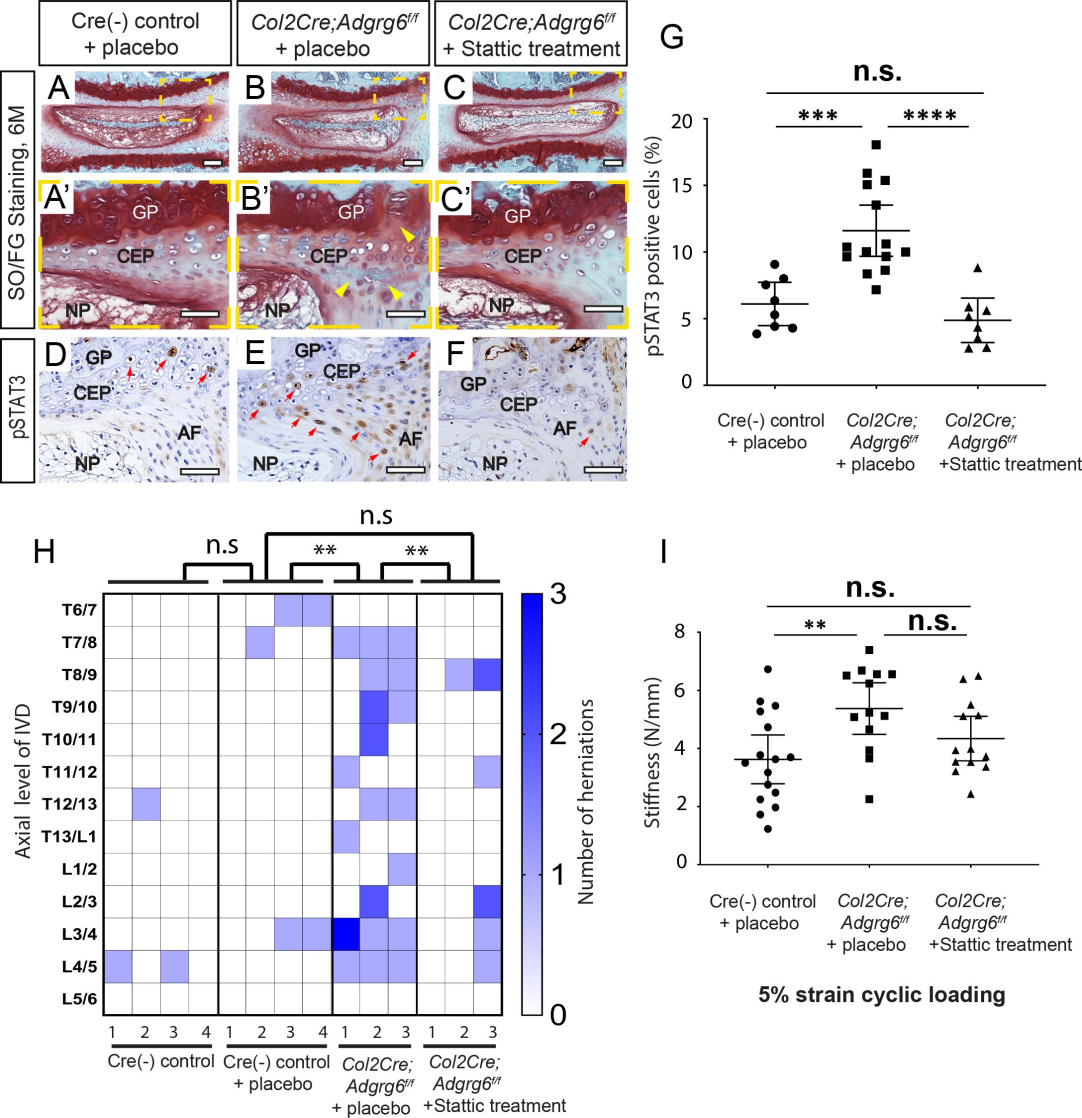
Inhibition of STAT3 by Stattic alleviates the formation of disc herniations and disc stiffness attributed to loss of ADGRG6 function. (A-C’) Representative Safranin-O/Fast green staining and (D-F) pSTAT3 IHC in medial-sectioned mouse IVD from placebo-treated Cre (-) control (A-A’, D), placebo-treated *Col2Cre;Adgrg6*^*f/f*^ mutant (B-B’, E), and Stattic treated *Col2Cre;Adgrg6*^*f/f*^ mutant mice (C-C’, F) by the age of 6 months (A-C’) or 2 months (D-F) (*n* = 3 mice for each group). *Col2Cre;Adgrg6*^*f/f*^ mutant mice display defects of the IVD including lesions and clefts in the CEP and GP (yellow arrowheads, B’) and increased, expression of pSTAT3 in CEP and AF (red arrowheads, E), which is reduced by Stattic treatment (C-C’, F). (G) Quantification of positive pSTAT3 cells in Cre (-) control or in *Col2Cre;Adgrg6*^*f/f*^ mutant mice mutant mouse IVDs by the age of two months after two weeks of Stattic treatment. (*n* = 3 mice for each group, two to five IVDs were scored for each mouse. Dots plot with mean ± SD. ***p≤0.001, ****p≤0.0001, One way ANOVA followed by Tukey HSD test. n.s, not significant.) (H) Heat map to represent contrast-enhanced microCT data from 6-month-old mice from three experimental groups: four placebo-treated Cre (-) controls; three placebo-treated *Col2Cre;Adgrg6*^*f/f*^ mutants; and three Stattic-treated *Col2Cre;Adgrg6*^*f/f*^ mutants. Plotted by the axial level of the IVD (left axis) and the number of herniations (right axis) observed in each mouse. (**p≤0.01, One way ANOVA followed by Tukey HSD test. n.s, not significant.) (I) Mechanical testing on lumbar discs isolated from the same samples imaged in (H) using 5% strain cyclic loading. (Three to five IVDs were analyzed /mouse. Dots plot with mean ± 95% CI. **p≤0.01, One way ANOVA followed by Tukey HSD test. n.s, not significant.) Scale bars: 200μm in (A-C), 50μm in (A’-C’) and (D-F). *AF- annulus fibrosis*, *CEP- cartilaginous endplate*, *GP- growth plate*, *and NP- nucleus pulposus*.

Importantly, these constitutive conditional *Col2Cre;Adgrg6*^*f/f*^ mutant mice display no obvious histopathology of IVD and growth plate at P20 (S7B and S7B’ Fig), yet exhibit adult-onset endplate-oriented herniations by histology at 8 months (S7D and S7D’ Fig), while the annulus fibrosus and nucleus pulposus is unremarkable when visualized at the midline (S7E Fig). We set up a treatment regime where in both Cre (-) control and mutant groups would receive Stattic, a nonpeptidic STAT3 inhibitor (25mg/kg, dissolved in DMSO/PBS/Tween-20), [45], or placebo (DMSO/PBS/Tween-20) via i.p. injection for 16 weeks beginning at 1.5 months and imaged at 6 months. IHC staining of pSTAT3 and quantification analysis revealed that after two weeks of Stattic treatment, the percentile of pSTAT3 positive cells was significantly reduced in the Stattic treated mutant mice (Fig 6F and 6G) compared with the placebo treated mutant mice (Fig 6E and 6G), but was not distinguishable from the placebo treated Cre(-) control mice (Fig 6D and 6G), demonstrating efficient inhibition of the STAT3 signaling by systemic Stattic treatment.

By contrast-enhanced μCT imaging of the placebo treated *Col2Cre;Adgrg6*^*f/f*^ mutant mice at 6 months, we quantified an average of 9.6 herniations/mouse (*n* = 3 mice; 29 total herniations from 39 total IVDs imaged) (Fig 6H). In this background, Cre (-) littermate controls displayed rare occurrences of endplate-oriented herniations in both the placebo-injected (*n* = 4 mice; 5 total herniations from 52 total IVDs imaged) and uninjected (*n* = 4 mice; 3 total herniations from 52 total IVDs imaged) groups (Fig 6H). Importantly, we observed a decrease in the incidence of endplate-herniations in *Col2Cre;Adgrg6*^*f/f*^ mutant mice treated with Stattic for 16-weeks (*n* = 3 mice; 9 total herniations from 39 total IVDs imaged) (Fig 6H). One way ANOVA followed by Tukey HSD test indicated that the mean score for the Stattic-treatment condition ((M)ean = 0.23, SD = 0.54) was significantly different (*p*<0.01) than the placebo-treated *Col2Cre;Adgrg6*^*f/f*^ mutant mice (M = 0.74, SD = 0.56), but did not differ significantly from the placebo-treated Cre (-) control littermates (M = 0.096, SD = 0.09) (S3 Table). Post-hoc analysis shows a medium effect size (Cohen’s δ: 0.7859) comparing the Stattic treatment and placebo *Col2Cre;Adgrg6*^*f/f*^ mutant mice (S3 Table). Given that 100% of *Col2Cre;Adgrg6*^*f/f*^ mutant mice displayed endplate-oriented herniations at 8 months, an a priori power analysis (assuming effect size 0.5; power of 0.99) suggested that we would need to image 40 discs per group to see a significant effect with a power of 0.99 between any two groups.

To understand if Stattic treatment also improve the biomechanical properties, we isolated lumbar discs (L1/2-5/6) from the same mice after contrast-enhanced μCT imaging for dynamic mechanical testing (*n* = 4 for placebo-treated Cre (-) controls, 16 discs; *n* = 3 for placebo-treated *Col2Cre;Adgrg6*^*f/f*^ mutants, 13 discs; and *n* = 3 for Stattic-treated *Col2Cre;Adgrg6*^*f/f*^ mutants, 13 discs). We demonstrated that, under 5% strain cyclic loading, placebo-treated *Col2Cre;Adgrg6*^*f/f*^ mutant IVDs showed significant increase in the stiffness (5.37±1.43 N/mm) compared with the placebo-treated Cre (-) controls (3.63±1.58 N/mm) (Fig 6I, p<0.01, One way ANOVA followed by Tukey HSD test). Importantly, after 16 weeks of Stattic treatment, the stiffness of the Stattic-treated *Col2Cre;Adgrg6*^*f/f*^ mutant IVDs reduced to 4.34±1.27 N/mm, which is not statically significant from neither the placebo-treated Cre (-) control group, nor the placebo-treated *Col2Cre;Adgrg6*^*f/f*^ mutant group (Fig 6I, One way ANOVA followed by Tukey HSD test), suggesting a partial rescue of the increased stiffness in the *Col2Cre;Adgrg6*^*f/f*^ mutant IVDs after Stattic treatment. We also performed a correlation analysis (Pearson’s r) of the dynamic mechanical testing data and the contrast-enhanced μCT imaging generated from the same lumbar discs in these three experimental group. We found no significant correlation between these variables in individual disc (S12 Fig). This could be due to the limitation that the mechanical testing was only reliable for the lumbar discs [21].

We observed increased pSTAT3 expression in the placebo-treated *Col2Cre;Adgrg6*^*f/f*^ mutant mice compared to Cre (-) control mice treated with placebo (p≤0.001) (Fig 6D, 6E and 6G). Importantly, Stattic treatment was observed to decrease the number pSTAT3 positive cells in the IVD of *Col2Cre;Adgrg6*^*f/f*^ mutant mice (p≤0.0001) (Fig 6F and 6G), comparable to the incidence observed in Cre (-) littermate control IVDs. Next, we assayed several other markers of degenerative IVD in these Stattic-treatment experimental groups. We found that COLX expression in the CEP was reduced in Stattic-treated *Col2Cre;Adgrg6*^*f/f*^ mutant mice compared with the placebo-treatment groups (S11A–S11C’ Fig). In contrast, Stattic treatment had little effect on the general reduction of COLII and SOX9 expression observed in *Col2Cre;Adgrg6*^*f/f*^ mutant mice, similar results were observed by qPCR analysis of gene expression in Stattic-treated *Adgrg6* KO ATDC5 cells undergoing maturation (S11J Fig). These data suggest that additional effectors of ADGRG6 function, apart from STAT3 signaling, are required for maintenance of normal gene expression profiles in CEP and growth plate.

Taken together, our studies demonstrated that dysregulation of STAT3 signaling is associated with endplate-oriented herniations in the IVD of *Adgrg6* conditional mutant mice. Blockade of STAT3 signaling by Stattic treatment can protect against the formation of the endplate-oriented herniations in these mutant mice, potentially through a restoration of normal biomechanical properties of the disc. These results demonstrate that dysregulation of STAT3 signaling can drive endplate-oriented disc herniation, and ADGRG6/STAT3 signaling is a promising therapeutic target for degenerative joint disorders.

## Discussion

In this study, we establish for the first time that ADGRG6 signaling acts as a positive regulator of homeostatic mechanisms of the CEP and growth plate. Loss of homeostasis is observed based on global changes in gene expression in the *Adgrg6* mutants IVD tissues including: alterations of several anabolic and catabolic factors, increased expression of collagens associated with fibrotic and degenerative cartilage tissues, and increased STAT3 activation. Histological analysis of several of these factors showed altered expression specifically in the CEP and growth plate. We further demonstrate that alterations of protein and gene expression in the IVD and growth plate and increased mechanical stiffening of the discs occur during early postnatal development (P20-1.5M), several months prior to obvious histopathology and endplate-oriented herniations observed in adult mice. We suggest that these transcriptional changes coupled with mild increases in cell death lead to a general stiffening of the IVD, which in turn synergistically contributes to the formation of the endplate-oriented disc herniation in adult mice. Finally, we show that inhibition of STAT3 activation provides a protective effect against the formation of the endplate-oriented disc herniations in ADGRG6 mutant discs and growth plate tissues. This is important given the positive association of increased STAT3 signaling and multiple degenerative joint diseases, including disc degeneration [46], disc herniation [47], and osteoarthritis [48] in humans.

We demonstrate that ADGRG6 acts as a cell autonomous regulator of the STAT3 signaling in vivo and in cartilagenous cell culture. The role in vivo of ADGRG6 appears to be most specific to the CEP and growth plate, as other IVD tissues were not greatly affected even out to 8-months of age. However, a more direct test of the specific role of *Adgrg6* in the nucleus pulposus or annulus fibrosus tissues of the IVD is warranted. It will also be interesting to test if blockade of STAT3 signaling has a general protective effect of CEP integrity, not dependent on ADGRG6 function. However, to our knowledge there is not a well-established alternative experimental mouse model of endplate-oriented disc herniations to test this. Finally, it will be important to determine how STAT3 signaling contributes to histopathology observed in other models of disc degeneration including models with lateral herniations and/or pathology of the nucleus pulposus or annulus fibrosus.

The role of ADGRG6 appears to be largely dispensable for embryonic and early postnatal developmental of the IVD, yet is critical for postnatal mechanisms of homeostasis. We demonstrate that ADGRG6 is important for maintaining normal expression of SOX9 in vivo and in chondrogenic ATDC5 cell culture, where we observed reduced expression of *Sox9* along with its direct target gene *Col2a1* in *Adgrg6* KO cells. SOX9 is a master transcription factor for both chondrogenesis during embryonic development [49] and cartilage maintenance during postnatal development [50], in the IVD. Regulation of *Adgrg6* and *Sox9* was also reported in the cartilaginous semicircular canal which similarly display altered expression of several extracellular matrix genes, as well as, decreased expression of *sox9b* in *adgrg6/gpr126* mutant zebrafish [10]. Genetic ablation of *Sox9* in cartilaginous tissues in adult mice leads to reduction of *Adgrg6/Gpr126* expression, as well as many pathological similarities of the IVD as we described in this work, including increased cell death and alterations of extracellular matrix gene expression [50]. In contrast, *Sox9* mutant mice exhibit more severe IVD defects including decreased disc height and loss of proteoglycan staining. This differences in pathology may be explained by an incomplete or perhaps more gradual loss of SOX9 expression in the conditional *Adgrg6* mouse models presented here, which may stimulate undefined compensatory mechanisms [51]. These data suggest that ADGRG6 and SOX9 have some degree of co-regulation in cartilaginous tissues. The identification of factors that govern this regulation and how this mechanistically contributes during homeostasis and disease warrants further investigation.

We previously demonstrated a critical role for *Adgrg6* in the formation of scoliosis in young mice (onset at around P20-P40) [11]. Here we demonstrate a novel defect of endplate-oriented disc herniations in both *Col2Cre;Adgrg6*^*f/f*^ and *ATC;Adgrg6*^*f/f*^ adult mutant mouse (at 6 to 8 months of age). The incidence of scoliosis in *Col2Cre;Adgrg6*^*f/f*^ is ~80%, while only ~12% of *ATC;Adgrg6*^*f/f*^ mutant mice display spine curvatures. In contrast, endplate-oriented herniations were observed in ~100% of conditional *Adgrg6* mutant mouse models. The localization of post-natal onset scoliosis in both models is localized to the thoracic spine, while the formation of endplate-oriented herniations is observed in both thoracic and lumbar sections of the spine. From this, we suggest that the mechanisms promoting the formation of endplate-oriented disc herniations in adult mice are partially independent from the pathogenesis of postnatal-onset idiopathic scoliosis in these mutant mice. However, it is tempting to speculate that alterations of the mechanical properties of the IVD during postnatal development underlie the susceptibility of scoliosis during early postnatal development and may also promote endplate-oriented herniations in adults as well. Additional ongoing studies are seeking understand the cellular pathogenesis underlying *Adgrg6*-dependent scoliosis and how spine curvature is related to molecular changes in the CEP and growth plate *Adgrg6* mutant mice.

Using an innovative contrast-enhanced μCT imaging approach of the intact mouse spine, we for the first time describe the appearance and distribution of the endplate-oriented herniations in mice. Using this approach, we also observed a low frequency of endplate-oriented disc herniations in some Cre(-) control mice suggesting variable genetic susceptibility of this adult-onset pathology in mouse. Standard histology can easily miss subtle defects of the IVD, as we illustrate in adjacent sections from several independent *Adgrg6* conditional mutant mice (S2 and S4 and S7 Figs). Given the high resolution of the contrast-enhanced μCT imaging and ease of imaging the intact mouse spine we suggest this technique is a superior method for the identification of age-related defects of the IVD. In the future, it will be interesting to apply comprehensive contrast-enhanced μCT imaging to determine how prevalent endplate defects are in a variety of inbred strains during aging.

**Fig 7:**
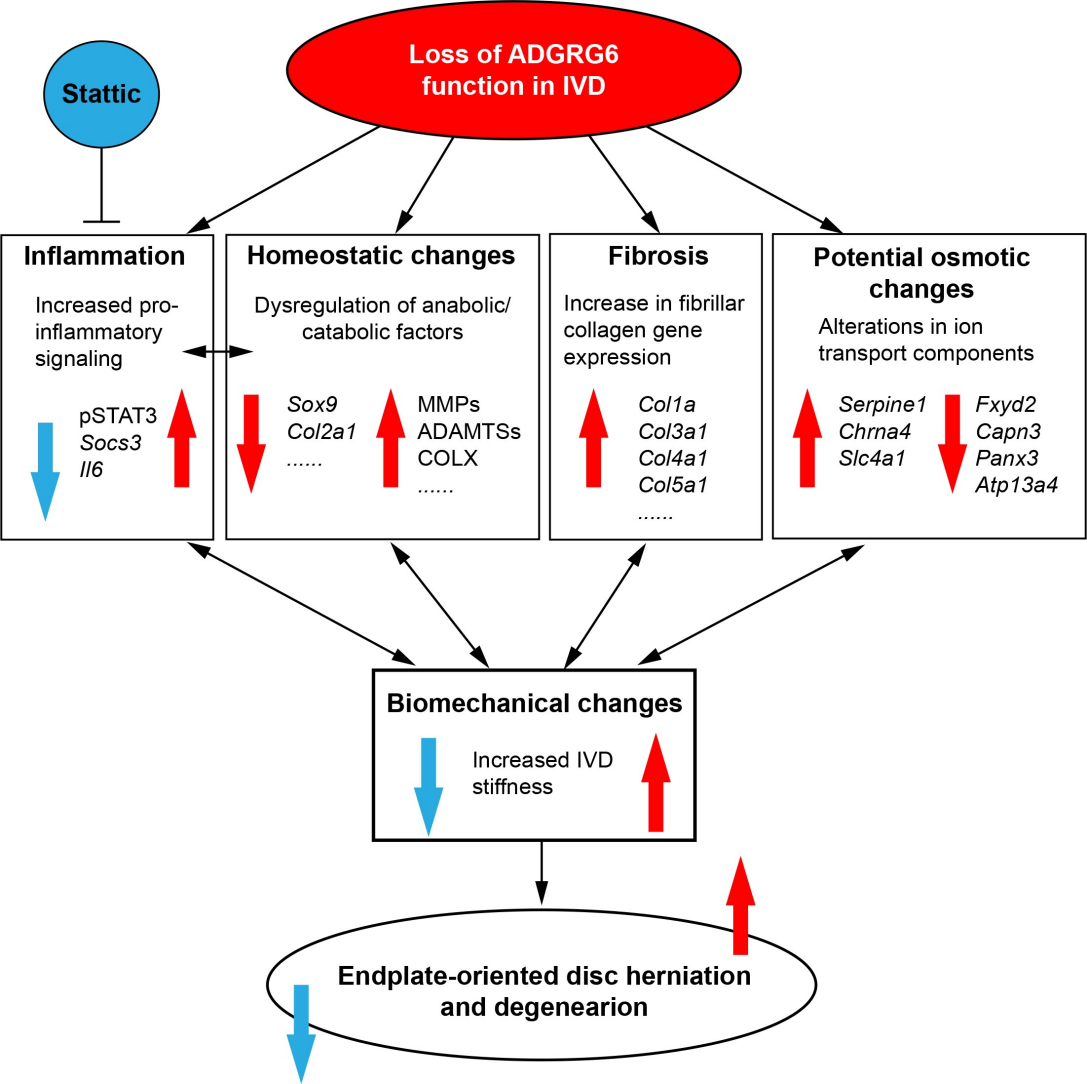
A model of ADGRG6 in regulating IVD integrity. Loss of ADGRG6 function in cartilaginous tissues of the IVD leads to increased pro-inflammatory singling (especially STAT3 signaling), dysregulation of anabolic and catabolic factors, fibrosis of the disc, and potential osmotic changes of the disc in young mice. These changes synergistically weaken the IVD and result in altered biomechanical changes (increased disc stiffness), and ultimately leads to endplate-oriented disc herniation in adult mice. Inhibition of STAT3 signaling by Stattic treatment can alleviates the formation of disc herniations and partially rescue the disc stiffness upon loss of ADGRG6 function.

Our findings support a model where increased disc stiffness in young *Adgrg6* mutant mice proceed the onset of endplate-oriented disc herniations. We demonstrate the upregulation of multiple collagen genes associated with fibrosis in postnatal mice (P20), which is expected to result in disturbed stress distribution of the IVD and concentrate loading at the CEP. This shift in disc mechanics is postulated to increase the risk of CEP fractures, decreases nutrient supply, and the lead to the formation of disc herniations [6, 52–54]. Several studies have shown that different combinations of mechanical loads (e.g. torsion, rotation, and flexion) applied to the disc lead to peak strain locations at both disc-bone interface and in the annulus fibrosus, which can ultimately lead to disc failure [55–57] and suggests that defects in the CEP and growth plate in mice may increase the risk for endplate-oriented disc herniation. Moreover, the adult IVD is thought to be an avascular tissue, as such, its major source of nutrient flux occurs via diffusion through the CEP [58]. As such, CEP sclerosis and reduced nutrient supply in the IVD is correlated with increased expression of collagen genes associated with fibrosis, catabolic enzymes, and expression of COLX [59, 60]. In this way, a viscous cycle comprising increased expression of catabolic enzyme activity, pro-inflammatory factors, and apoptosis, can further exacerbate the integrity of the IVD leading to endplate-oriented herniation during adult development [6, 54]. In conclusion, we suggest that changes of typical collagen expression in the disc coupled with increased expression of other catabolic factors such as MMPs and ADAMTSs, act synergistically to weaken the CEP and growth plate and contribute to the stiffness of the IVD and ultimately increase the susceptibility of the endplate-oriented disc herniation in this mouse model (Fig 7).

We also demonstrate an upregulation of STAT3 signaling in *Adgrg6* conditional mutant mice, prior to overt histopathology. Analysis of *Adgrg6* KO ATDC5 cells in culture demonstrate that the activation of the IL-6/STAT3/*Socs3* pathway is an intrinsic property of cartilaginous cells and that this is regulated by ADGRG6 function. Increased expression of IL-6 and activation of STAT3 (pSTAT3) has been observed in human patients with disc degeneration and disc herniation [38, 61]; circulating IL-6 is positively associated with radiographic osteoarthritis and loss of knee cartilage loss in humans [62]; and IL-6/STAT3 signaling is activated in trauma-induced osteoarthritis [63, 64]. Here we show that systemic inhibition of STAT3 signaling with Stattic [45] acts to alleviate the onset and progression of endplate-oriented herniations of the IVD, which was recently also demonstrated to improve joint remodeling in a post-traumatic osteoarthritis model in mouse [64]. Together these studies underscore the need to further elaborate on the role of STAT3 activation for the initiation and progression of other degenerative joint disorders. Altogether, this study provides multiple supporting evidence of the regulatory role and therapeutic value of ADGRG6/STAT3 signaling for the onset and progression of endplate-specific disc defects.

## Materials and methods

### Ethics statement

All animal research was conducted according to federal, state, and institutional guidelines and in accordance with protocols approved by Institutional Animal Care and Use Committees at University of Texas at Austin (AUP-2018-00276).

### Mouse strains

Mice were housed in standard cages and maintained on a 12-hour light/dark cycle, with rodent chow and water available ad libitum. All mouse strains were described previously, including *Adgrg6*^*f/f*^ (Taconic #TF0269) [65]; *Rosa26;LacZ* (B6;129S-*Gt(ROSA)26Sor*/J) [66]; *ATC* [13], and *Col2Cre* [67]. Doxycycline (Dox) was administered to *ATC; Adgrg6*^*f/f*^ mice and littermate controls with two strategies: (i) inducing from embryonic day (E)0.5-postnatal day (P)20 by ad libitum feeding of Dox-chow (Test Diet, 1814469) to plugged isolated females, and supplemented with intraperitoneal (IP) injections of the pregnant dames once/week (10mg Dox/kg body weight) throughout the pregnancy until the pups were weaned at P20; (ii) inducting from P1-P20 by ad libitum feeding of Dox-chow to the mothers after the pups were born, and supplemented with intraperitoneal (IP) injections of the mothers once/week (10mg Dox/kg body weight) until the pups were weaned at P20. *ATC; Rosa-LacZ*^*f/+*^ mice were induced with the same strategies. STAT3 inhibitor Stattic (25mg/kg, dissolved in DMSO) or placebo (DMSO/PBS/Tween-20) were administered to *Col2Cre; Adgrg6*^*f/f*^ mutant mice or Cre (-) littermate controls via i.p. injection once/week for 16 weeks beginning by the age of 1.5 months. Mice were harvested at P2, P20, 1.5 months, 6 months and 8 months of age.

### Analyses of mice

Histological analysis was performed on thoracic spines fixed in 10% neutral-buffered formalin for 3 days at room temperature followed by 1-week decalcification in Formic Acid Bone Decalcifier (Immunocal, StatLab). After decalcification, bones were embedded in paraffin and sectioned at 5μm thickness. Safranin O/Fast Green (SO/FG) and Alcian blue Hematoxylin/Orange G (ABH/OG) staining were performed following standard protocols (Center for Musculoskeletal Research, University of Rochester). Immunohistochemical analyses were performed on paraffin sections with traditional antigen retrieval and colorimetric development methodologies with the following primary antibodies: anti-Collagen II (Thermo Scientific, MS235B), anti-Collagen X (Quartett, 1-CO097-05), anti-SOX9 (Santa Cruz Biotechnology, sc-20095), anti-Lubricin (PRG4) (Abcam, ab28484), anti-MMP-13 (Thermo Scientific, MS-825-P), anti-IL-6 (Abcam, ab6672), and anti-phospho-STAT3 (Cell Signaling, #9145). The Terminal deoxynucleotidyl transferase dUTP Nick-End Labeling (TUNEL) cell death assay was performed on paraffin sections with the In Situ Cell Death Detection Kit, Fluorescein (Roche) according to the manufacturer’s instructions. The b-galactosidase staining was performed on frozen sections as previously described [63]. Spines were harvested and fixed in 4% paraformaldehyde for 2 hours at 4°C and decalcified with 14% EDTA at 4°C for 1 week. Tissues were washed in sucrose gradient, embedded with Tissue-Tek OCT medium, snap-frozen in liquid nitrogen, and sectioned at 10μm with a Thermo Scientific HM 550 cryostat. *In situ* hybridization using a Digoxygenin-labeled antisense riboprobe for *Adgrg6* was performed on 5μm paraffin sections as described previously with modifications [11], and detected with either a chromogenic substrate (BM Purple, Roche) or a tyramine-amplified fluorescent antibody (Perkin Elmer).

### Cell culture

ATDC5 cells (Sigma, 99072806) were maintained in DMEM/F-12 (1:1) medium (Gibco, 11330032) supplemented with 5% FBS and 1% penicillin/streptomycin. ATDC5 cells were maturated in DMEM/F-12 (1:1) medium supplemented with 5% FBS, 1% penicillin/streptomycin, 1% ITS premix (Corning, 354352), 50μg/ml ascorbic acid, 10nM dexamethasone, and 10ng/ml TGF-β3 (Sigma, SRP6552) for 5, 10, and 15 days.

Both wild type and *Adgrg6* KO ATDC5 cells were treated with 100ng/ml recombinant human IL-6 protein (rIL-6) (R&D System, 206-IL) for 2 hours before protein extraction. Both wild type and *Adgrg6* KO ATDC5 cells were treated with 10nM Stattic in maturation medium for 10 days before RNA extraction.

### Generation of the *Adgrg6* KO cell line

CRISPR reagents were generated to target the 3rd exon of mouse *Adgrg6* (ENSMUST00000041168.5) using the following oligos: *Adgrg6*-g33-fwd ACACCGAGGGTAACACGGAGACGTAAG and *Adgrg6*-g33-rev AAAACTTACGTCTCCGTGTTACCCTCG and cloned into a lentiviral packing vector (lentiCRISPR v2 was a gift from Feng Zhang (Addgene plasmid # 52961)) along a pCas9_GFP (a gift from Kiran Musunuru (Addgene plasmid # 44719)). Lentiviral particle packaging was in A293T cells using standard 3rd generation approach (https://tronolab.epfl.ch/page-148635-en.html). Human embryonic kidney (HEK) 293T cells (Sigma) were maintained in DMEM supplemented with 10% fetal bovine serum, 2mM GlutaMAX (Life Technologies), 100U/ml penicillin, and 100ug/mL streptomycin at 37°C with 5% CO_2_ incubation. 293T cells were seeded into 6cm plates (Corning) one day prior to transfection at a density of 2x10^6^ cells per well. 293T cells were transfected using FUGENE 6 (Promega) following the manufacturer’s recommended protocol. For each plate, a total of 0.5ug of each plasmid was used. At 2 and 4 days post transfection, the cell media was collected and filtered with 0.45 μM filter (Corning) and stored at -80°C.

ATDC5 were plated in 6-well plates to 80% confluency and lentiviral transduction was using diluted viral media with 0.1% polybrene (EMD Millipore) for 24 hours followed by selection with 4μg/ml Blastocidin and Puromycin for 5 days post transfection. Serial dilution under selection was used to identify individual clones, expanded colonies were screened for INDEL mutations using *Adgrg6*-ex3-fwd—TTGACAGTTACTGCTTGATGCCCCC and *Adgrg6*-ex3-rev- CCCTTGGCAGTCGCTCCACAGAATT primers and amplicons were screen by Sanger sequencing to identify homozygous clones.

### RNA isolation and qPCR

Entire intervertebral discs from the thoracic and lumbar spine (T8-L5) of the 1.5-month old *ATC; Adgrg6*^*f/f*^ and control mice were isolated in cold PBS, snap frozen and pulverized in liquid nitrogen. Total RNA from intervertebral discs was isolated using the TRIzol Reagent (Invitrogen, 15596026), and cleaned up with the Direct-zol RNA miniprep kit (Zymo Research, Z2070). Total RNA of the cultured ATDC5 cells was isolated using the RNAeasy mini kit (Qiagen, 74104). Reverse transcription was performed using 1μg total RNA with the iScript cDNA synthesis kit (BioRad). Reactions were set up in technical and biological triplicates in a 96 well format on a BioRAD CFX96 real-time PCR detection system, using SYBR green chemistry (SsoAdvanced, BioRad). The PCR conditions were 95°C for 3 min followed by 40 cycles of 95°C for 10s and 58°C for 30s. Gene expression was normalized to *b-actin* mRNA and relative expression was calculated using the 2^-(ΔΔCt)^ method. All qPCR primers sequences are listed in S4 Table. PCR efficiency was optimized and melting curve analyses of products were performed to ensure reaction specificity.

### RNA isolation, library construction and sequencing

Entire intervertebral discs from the thoracic and lumbar spine (T8-L5) of the P20 *Col2Cre; Adgrg6*^*f/f*^ and control mice were isolated in cold PBS, snap frozen and pulverized in liquid nitrogen. Total RNA was extracted using Trizol reagent (Invitrogen, CA, USA) following the manufacturer's procedure. The total RNA quantity and purity were analyzed on Bioanalyzer 2100 and RNA 6000 Nano LabChip Kit (Agilent, CA, USA) with RIN number >7.0. Total RNA was subjected to isolate Poly (A) mRNA with poly-T oligo attached magnetic beads (Invitrogen). RNA fragments were reverse-transcribed to create the final cDNA libraries following the NEBNext Ultra RNA Library Prep Kit (Illumina, San Diego, USA), paired-end sequencing was performed. All raw reads are available as GSE128402 in the NCBI Gene Expression Omnibus.

### Bioinformatics analysis

#### Transcripts assembly

Cutadapt [68] and perl scripts in house were used to remove the reads that contained adaptor contamination, low quality bases and undetermined bases. Then sequence quality was verified using FastQC (http://www.bioinformatics.babraham.ac.uk/projects/fastqc/). We used HISAT2 [69] to map reads to the genome of Mus Musculus (GRCm38.88). The mapped reads of each sample were assembled using StringTie [70]. Then, all transcriptomes from biological samples were merged to reconstruct a comprehensive transcriptome using perl scripts and gffcompare. After the final transcriptome was generated, StringTie [71] and Ballgown [70] was used to estimate the expression levels of all transcripts.

#### Different expression analysis of mRNAs

StringTie [71] was used to perform expression level for mRNAs by calculating FPKM. The differentially expressed mRNAs were selected with log2 (fold change) >1 or log2 (fold change) <-1 and with statistical significance (p value < 0.05) by R package Ballgown [70].

### Western blotting

For western blotting analysis, total proteins were extracted from cells with protein extraction buffer [50mM HEPES, 1.5mM EDTA (pH 8.0), 150mM NaCl, 10% glycerol, 1% Triton X-100] supplemented with protease and phosphatase inhibitors (Roche). 10mg of protein from each sample was resolved by 4–15% SDS-polyacrylamide gel electrophoresis and transferred to the nitrocellulose membranes. Western blots were then blocked with LI-COR blocking buffer and incubated overnight with primary antibodies anti-STAT3 (Cell Signaling, #4904), anti-pSTAT3 (Cell Signaling, #9145), and anti-GAPDH (Cell Signaling, #2118) at 4°C with gentle rocking. The next day western blots were detected with the LI-COR Odyssey infrared imaging system.

### Contrast-enhanced μCT imaging and segmentation

Samples undergoing contrast-enhanced micro-computed tomography (μCT) were blinded and incubated in a 35%w/v solution Ioversol in PBS (OptiRay 350, Guerbet, St. Louis) supplemented with 1% penicillin–streptomycin at 37C one day prior to scanning. Immediately prior to scanning, the sample was removed from the solution and wrapped in PBS-soaked gauze. These samples were mounted in 2% agarose gels and then scanned using the microCT40 system (Scanco Medical, CH) operating at 6 μm voxel size (45kVp, 177uA, 300 ms integration). Following our previous method for segmentation of murine IVDs [15, 72], the μCT CT data is exported as a DICOM file for further processing. Following an initial median filter (sigma = 0.8, support = 3), bone is then thresholded out, and the soft tissue not part of the IVD was removed by drawing contours around the outer edge of every five transverse slices of the AF and morphed using a linear interpolation. The remaining voxels are designated as the whole disc mask. From the masks of the whole disc, volumes and average attenuations (intensity) are calculated. The volume was determined from the total number of voxels contained within the mask and the attenuation is taken as the average 16-bit grayscale value of the voxels. Visualizations of the μCT were obtained using OsiriX (Pixmeo, Geneva). The volume of the contoured disc was then measured. Endplate defects were defined by three or more consecutive slices that had a rupture in the same area of the endplate. This method was chosen so as to eliminate any potential spatial artifacts that may be misidentified as ruptures.

### Mechanical testing

The mechanical properties of the isolated intervertebral discs were determined using dynamic compression on a microindentation system (BioDent 1000; Active Life Scientific, Santa Barbara, CA) with a 2.39 mm non-porous, flat probe [21]. The probe's load cell resolution is 0.001 N, and the system's Piezo actuator resolution is 0.01μm. Each sample was moved into position under the probe tip by gripping the aluminum platen. The indenter tip was aligned over each sample so that the probe covered the entire diameter of the disc. Each disc was first loaded sinusoidally at amplitude of 5% strain with a 10% peak strain at 1 Hz for 20 cycles with a 0.1 N preload. Stiffness for each disc was then calculated by averaging the loading slope of the 20 cycles. After the cyclic tests, the discs were monotonically overloaded to 50% strain at a loading rate of 10% strain per second. The loading slope value was obtained from the linear region of the force displacement curve from all loading curves. These samples were maintained in physiological PBS solution (pH 7.2) during and between trials to simulate the osmotic pressures found in the body and maintain hydration of the IVD.

### Quantification of collagen and proteoglycans

The wet weight of each isolated disc was taken after mechanical testing utilizing an analytical balance (A-200DS; Denver Instrument Company, Bohemia, NY). Samples were first digested in papain at 65°C for 18 h. The samples were then centrifuged and the supernatant collected and then plated in triplicate. Proteoglycan content was quantified using the colorimetric dimethyl-methylene blue (DMMB) assay [73] by measuring the absorbance 525nm with chondroitin sulfate from bovine cartilage as standards (Sigma-Aldrich, St. Louis, MO), and then normalized to wet weight of the IVD. The remaining papain-digested lysates were then used for hyproxyproline quantification. The amount of collagen was approximated by assuming that hydroxyproline accounts 1/7 of the mass of collagen. The samples were hydrolyzed in 12 N hydrochloric acid at 120°C for 3 h. The hydrolyzed samples were then plated in triplicates. A chloramine T colorimetric assay [74] and standardized using hydroxyproline by quantifying the absorbance at 560 nm using a plate reader (SpectraMax M2, Molecular Devices, Sunnyvale, CA).

### Statistics

Statistical analyses to compared the mutant and control groups were performed using 2-tailed Student’s *t*-test and one-way ANOVA followed by Turkey HSD test as appropriate (GraphPad Prism 7). Power analysis was done using G*Power. A p value of less than 0.05 is considered statistically significant.

## Supporting information

S1 Fig *In situ* expression of *Adgrg6* in the spine. (A, B) *In situ* hybridizations of *Adgrg6* in spine tissue (8 months) using Alkaline phosphatase/BM purple chromogenic developing shows strong *Adgrg6* expression (blue stain) in the growth plate (GP) and minor expression in the annulus fibrosis (AF) (red arrowheads) that is mostly abolished in *ATC;Adgrg6*^*f/f*^ mutant tissues (B); or using (C, D) tyramine-amplification fluorescence which shows expanded expression throughout the IVD including GP, CEP, AF, and NP, which is mostly diminished in *ATC;Adgrg6*^*f/f*^ mutant tissues. Robust expression was detected in periosteum of the long bone tissues in both control and the *ATC;Adgrg6*^*f/f*^ mutant moues (E, F, yellow arrows). (Induced from P1-P20, *n* = 3 for each group.) Scale bars: 200μm in (A-D); 50μm in (E, F). *AF- annulus fibrosis*, *CEP- cartilaginous endplate*, *GP- growth plate*, *and NP- nucleus pulposus*, *Tb- trabecular bone*, *Cb-cortical bone*, *P-periosteum*.
(TIF)

S2 Fig Embryonic loss of *Adgrg6* lead to endplate-oriented herniations of the IVD in adult *ATC;Adgrg6*^*f/f*^ mutant mice. (A-C) Representative 8-month-old mouse IVDs (induced form E0.5-P20) stained with Safranin-O/Fast green (SO/FG) (*n* = 3 for controls and *n* = 6 for mutants). Endplate-oriented disc herniation is indicated with yellow arrows in B and B’. These herniations are very hard to be captured by histological analysis. C is an earlier midline section of an adjacent mutant IVD as shown in B, showing no overt histopathology. Scale bars: 100μm in (A-C); and 50μm in (A’, B’). *AF- annulus fibrosis*, *CEP- cartilaginous endplate*, *GP- growth plate*, *and NP- nucleus pulposus*.
(TIF)

S3 Fig β-galactosidase staining of LacZ-reporter mice induced with different strategies. More robust recombination (blue signal) in CEP, GP, and AF of the IVD was observed in the *Col2Cre; Rosa-LacZ* mouse (B, E) compared with the *ATC; Rosa-LacZ* mouse when induced from E0.5-P20 (A, C) and P1-P20 (D). Recombination in periosteum (B, E, red arrows) and the outmost AF layers of the IVD (B, E, black arrows) was observed only in the *Col2Cre; Rosa-LacZ* mouse but not the *ATC; Rosa-LacZ* mouse. Scale bars: 100μm in (A-E). *CEP- cartilaginous endplate*, *GP- growth plate*, *AF- annulus fibrosis*, *NP- nucleus pulposus*, *Tb- trabecular bone*, *and P- periosteum*.
(TIF)

S4 Fig Postnatal loss of *Adgrg6* in *ATC;Adgrg6*^*f/f*^ mutant mice leads to degenerative changes in the IVDs. (A-D’) Representative 4-month-old (A-B’) or 8-month-old (C-D’) mouse IVDs stained with Safranin-O/Fast green (SO/FG). (Induced from P1-P20. For A-B’, *n* = 3 for controls and *n* = 5 for mutants; for C-D’, *n* = 4 for each group). Minor growth plate erosion is observed by the age of four months in mutant mice (yellow arrowheads, B), while more severe endplate-oriented disc herniations were observed by the age of 8 months (yellow arrowheads, D). (B’) is an earlier midline sections of the same mutant IVD as shown in B. (D’) is a midline section of an adjacent mutant IVD as shown in D, showing no overt histopathology. (E-L) IHC analysis of 8-month-old Cre (-) Control and *ATC;Adgrg6*^*f/f*^ mutant mouse IVDs (induced from P1-P20). Several protein markers of IVD health and disease are affected in *ATC;Adgrg6*^*f/f*^ mutant IVD including decreased expression of healthy disc markers COLII and SOX9 (G, blue arrows), and increased expression of the hypertrophic marker COLX (F, red arrows) and extracellular matrix modifying enzyme MMP-13 (J, red arrows). (*n* = 3 for each group.) Scale bars: 100μm in (A-D’); 50μm in (E-L). *CEP*: *cartilaginous endplate*, *GP*: *growth plate*, *AF*: *annulus fibrosis*, *and NP*: *nucleus pulposus*.
(TIF)

S5 Fig Young *ATC;Adgrg6*^*f/f*^ mutant mice display degenerative alterations of protein expression in the IVD. Large scale images of IHC analysis shown in Fig 2. IHC analysis of common markers of degenerative disc. *ATC;Adgrg6*^*f/f*^ conditional mutant IVDs display reduced expression of markers of healthy disc: SOX9 (B), PRG4 (D), and COLII (H); and increased expression of extracellular matrix modifying enzymes MMP-13 (F), hypertrophic marker COLX (J). Scale bars: 100μm in (A-J). *AF- annulus fibrosis*, *CEP- cartilaginous endplate*, *GP- growth plate*, *and NP- nucleus pulposus*.
(TIF)

S6 Fig Young *ATC;Adgrg6*^*f/f*^ conditional mutant mice display increased apoptosis in the IVD. (A, B) TUNEL (red fluorescence) staining of 1.5-month-old *ATC;Adgrg6*^*f/f*^ mutants (B, white arrows) display increased TUNEL positive cells compared to Cre (-) control (A) mice. (C) Graph of the ratio of TUNEL positive cells to total cells (DAPI) (*n* = 3 for each group, three to five IVDs were analyzed/mouse. Bars represent mean ± SD. *p≤0.05, two-tailed Student's *t* Test). *AF- annulus fibrosis*, *CEP- cartilaginous endplate*, *GP- growth plate*, *and NP- nucleus pulposus*.
(TIF)

S7 Fig *Col2Cre;Adgrg6*^*f/f*^ mutant mice display endplate-herniation of the IVD in old mice. (A-B’) Representative medial-sectioned mouse IVDs stained with Alcian blue/Orange G of Cre (-) control (A and A') and *Col2Cre;Adgrg6*^*f/f*^ mutant (B, B') mice at P20 (*n* = 3 for each group). No overt histopathology was observed in mutant mice at this young age. (C-E) Representative mouse IVDs stained with Safranin-O/Fast green (SO/FG) of Cre (-) control (C and C') and *Col2Cre;Adgrg6*^*f/f*^ mutant (D, D', and E) mice by the age of 8 months (*n* = 3 for each group). Endplate-oriented herniations is indicated with yellow arrows. These herniations are very hard to be captured by histological analysis (D is out of the typical plane of section). E is an earlier midline section of the same mutant IVD as shown in D, showing no overt histopathology. Scale bars: 200μm in (A, B) and (C-E), and 50μm in (A’, B’) and (C’, D’). *AF- annulus fibrosis*, *CEP- cartilaginous endplate*, *GP- growth plate*, *and NP- nucleus pulposus*.
(TIF)

S8 Fig No macrophage infiltration was observed in *ATC;Adgrg6*^*f/f*^ mutant mouse IVD. (A-D) IHC analysis of macrophage marker shows no strong signal of CD68 in *ATC;Adgrg6*^*f/f*^ mutant mouse IVD at 1.5months (A, B), or 8 months of age (C, D), except for some background signal at the herniation site (red arrow, D). (Induced from E0.5-P20, *n* = 3 for each group.) Scale bars: 50μm in (A-D). *CEP- cartilaginous endplate*, *GP- growth plate*, *and NP- nucleus pulposus*.
(TIF)

S9 Fig *Adgrg6* regulates ATDC5 cell maturation. (A) Alcian blue staining on ATDC5 cell culture during the maturation process. (B) Expression profiles of *Adgrg6*, *Col2a1*, *Acan*, and *Sox9* during ATDC5 cell maturation. The expression level of *Adgrg6* was gradually increased alone with other chondrogenesis markers including *Col2a1*, *Acan*, and *Sox9*. (*n* = 3 biological replicates and representative result is shown. Bars represent mean ± SD. *p≤0.05, two-tailed Student's *t* Test). Scale bars: 100μm in A.
(TIF)

S10 Fig *Adgrg6* KO cells showed increased expression of apoptosis marker during maturation. (A, B) Immunofluorescence against cleaved-Caspace-3 (green) and DAPI staining (blue) and (C) quantification showing increased apoptosis in *Adgrg6* KO cells during maturation (10 days). (*n* = 3 biological replicates and representative result is shown. Bars represent mean ± SD. *p≤0.05, two-tailed Student's *t* Test). Scale bars: 50μm in (A, B).
(TIF)

S11 Fig Inhibition of STAT3 activation by Stattic leads to reduced COLX expression but does not affect the expression of COLII and SOX9 in *Col2Cre;Adgrg6*^*f/f*^ mutant mice. (A-C’) IHC analysis of COLX in 6-month-old Cre (-) control (A, A’) and *Col2Cre;Adgrg6*^*f/f*^ mutant mice with (C, C’) or without (B, B’) Stattic treatment. Ectopic COLX expression was observed in CEP of the mutant mice (red arrows, B’), which is rescued after Stattic treatment (C’). (D-I) IHC analysis of COLII and SOX9 in 6-month-old Cre (-) control (A, D) and *Col2Cre;Adgrg6*^*f/f*^ mutant mice with (F, I) or without (E, H) Stattic treatment. Reduced COLII and SOX9 expression was observed in *Col2Cre;Adgrg6*^*f/f*^ conditional mutant mice compared with Cre (-) control (blue arrows, D, G), but no obvious improvement was observed after Stattic treatment (F, I). (*n* = 3 for each group). (J) qPCR analyses revealed that *Col10a1* and *Sox9* expression in *Adgrg6* KO ATDC5 cells was partially rescued after Stattic treatment (10nM for 10 days), however the expression of *Col2a1* was not significantly changed (*n* = 3 biological replicates and representative result is shown. Bars represent mean ± SD. *p≤0.05, **p≤0.01, ***p≤0.001, ****p≤0.0001, One way ANOVA followed by Tukey HSD test. n.s, not significant.) Scale bars: 100μm in (A-C), and 50μm in (A’-I). *AF- annulus fibrosis*, *CEP- cartilaginous endplate*, *GP- growth plate*, *and NP- nucleus pulposus*.
(TIF)

S12 Fig Correlation analysis between number of herniation and disc stiffness in *Col2Cre;Adgrg6*^*f/f*^ mutant mice. Correlation analysis (Pearson’s r) between number of herniation and disc stiffness were performed on 6-month-old mice from three experimental groups: four placebo-treated Cre (-) controls; three placebo-treated *Col2Cre;Adgrg*^*f/f*^ mutants; and three Stattic-treated *Col2Cre;Adgrg*^*f/f*^ mutants as shown in Fig 6. Dots plotted by mean ± SD. No correlation was detected.
(TIF)

S1 Table Mouse models and analysis used in this study. (XLSX)

S2 Table Differential gene expression RNA-sequencing analysis from P20 IVD derived from *Col2Cre;Adgrg6*^*f/f*^ and Cre(-) wild-type littermates. (XLSX)

S3 Table One-way ANOVA and multiple comparison of the Cre (-) control and *Col2Cre; Adgrg6*^*f/f*^ (mutant) mice groups with placebo or Stattic treatment. (XLSX)

S4 Table qPCR primers used in this study. (XLSX)

S1 Movie Rotating rendered contrast-enhanced microCT image of a Cre (-) control mouse intervertebral disc. (MOV)

S2 Movie Rotating rendered contrast-enhanced microCT image of a *ATC;Adgrg6*^*f/f*^ mutant mouse intervertebral disc. (MOV)
