# Supplementary figures and images for: Dysregulation of STAT3 signaling is associated with endplate-oriented herniations of the intervertebral disc in Adgrg6 mutant mice
Source: PLoS Genet. 2019 Oct 25;15(10):e1008096. doi: 10.1371/journal.pgen.1008096 (PMC6834287; doi:10.1371/journal.pgen.1008096)

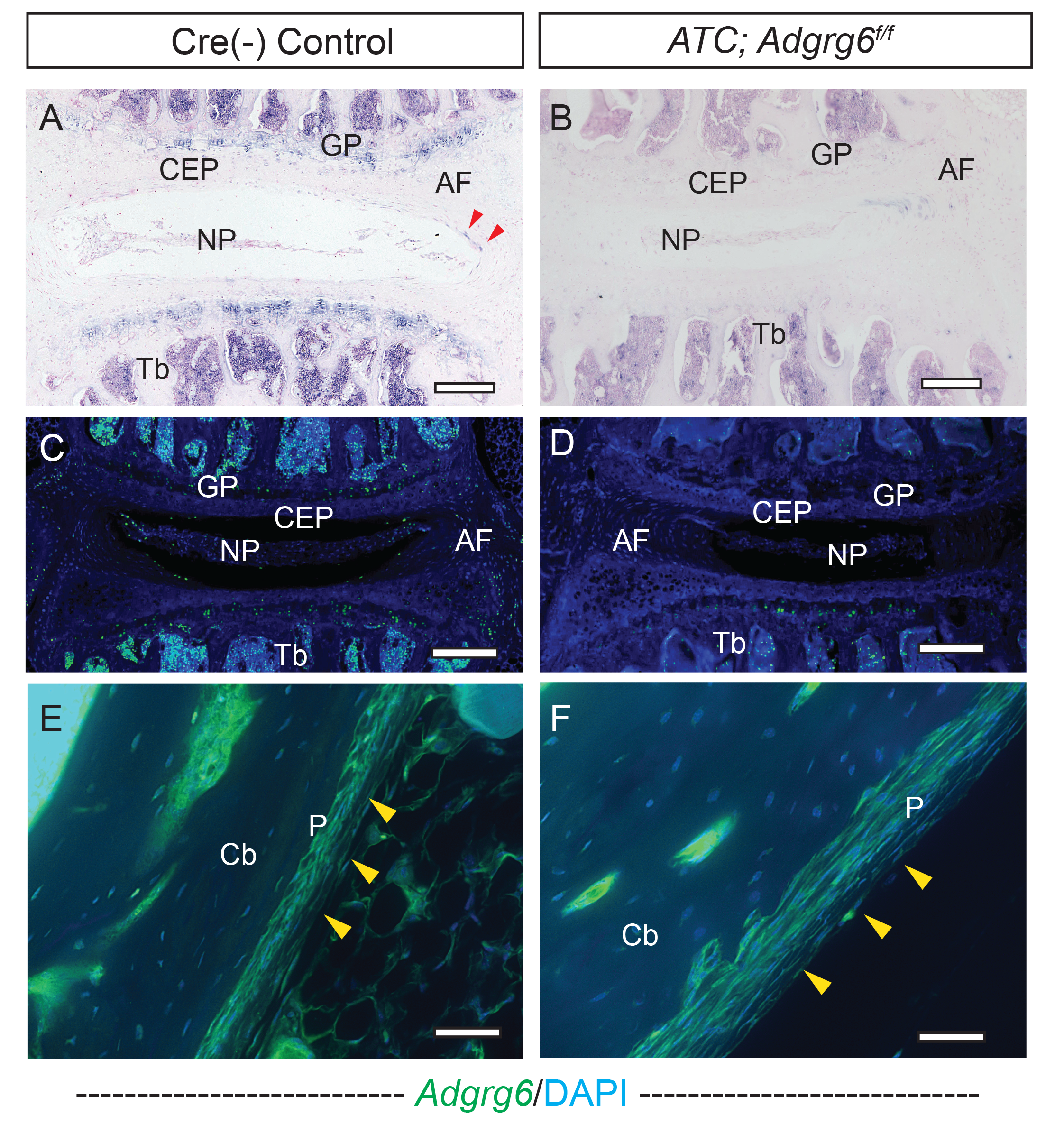

Supplement: S1 Fig — (A, B) In situ hybridizations of Adgrg6 in spine tissue (8 months) using Alkaline phosphatase/BM purple chromogenic developing shows strong Adgrg6 expression (blue stain) in the growth plate (GP) and minor expression in the annulus fibrosis (AF) (red arrowheads) that is mostly abolished in ATC;Adgrg6f/f mutant tissues (B); or using (C, D) tyramine-amplification fluorescence which shows expanded expression throughout the IVD including GP, CEP, AF, and NP, which is mostly diminished in ATC;Adgrg6f/f mutant tissues. Robust expression was detected in periosteum of the long bone tissues in both control and the ATC;Adgrg6f/f mutant moues (E, F, yellow arrows). (Induced from P1-P20, n = 3 for each group.) Scale bars: 200μm in (A-D); 50μm in (E, F). AF- annulus fibrosis, CEP- cartilaginous endplate, GP- growth plate, and NP- nucleus pulposus, Tb- trabecular bone, Cb-cortical bone, P-periosteum. (TIF) [file pgen.1008096.s001.tif]

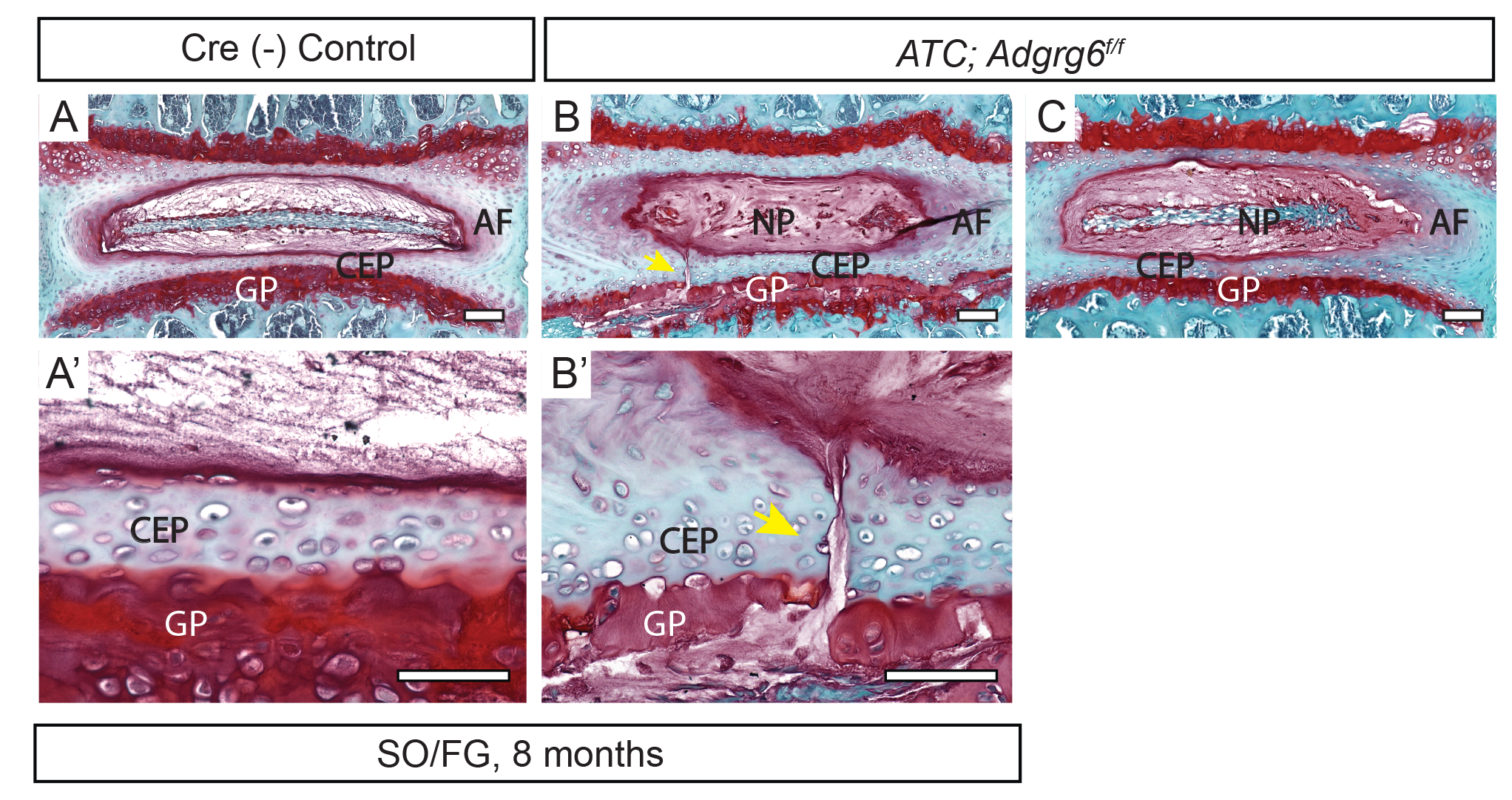

Supplement: S2 Fig — (A-C) Representative 8-month-old mouse IVDs (induced form E0.5-P20) stained with Safranin-O/Fast green (SO/FG) (n = 3 for controls and n = 6 for mutants). Endplate-oriented disc herniation is indicated with yellow arrows in B and B’. These herniations are very hard to be captured by histological analysis. C is an earlier midline section of an adjacent mutant IVD as shown in B, showing no overt histopathology. Scale bars: 100μm in (A-C); and 50μm in (A’, B’). AF- annulus fibrosis, CEP- cartilaginous endplate, GP- growth plate, and NP- nucleus pulposus. (TIF) [file pgen.1008096.s002.tif]

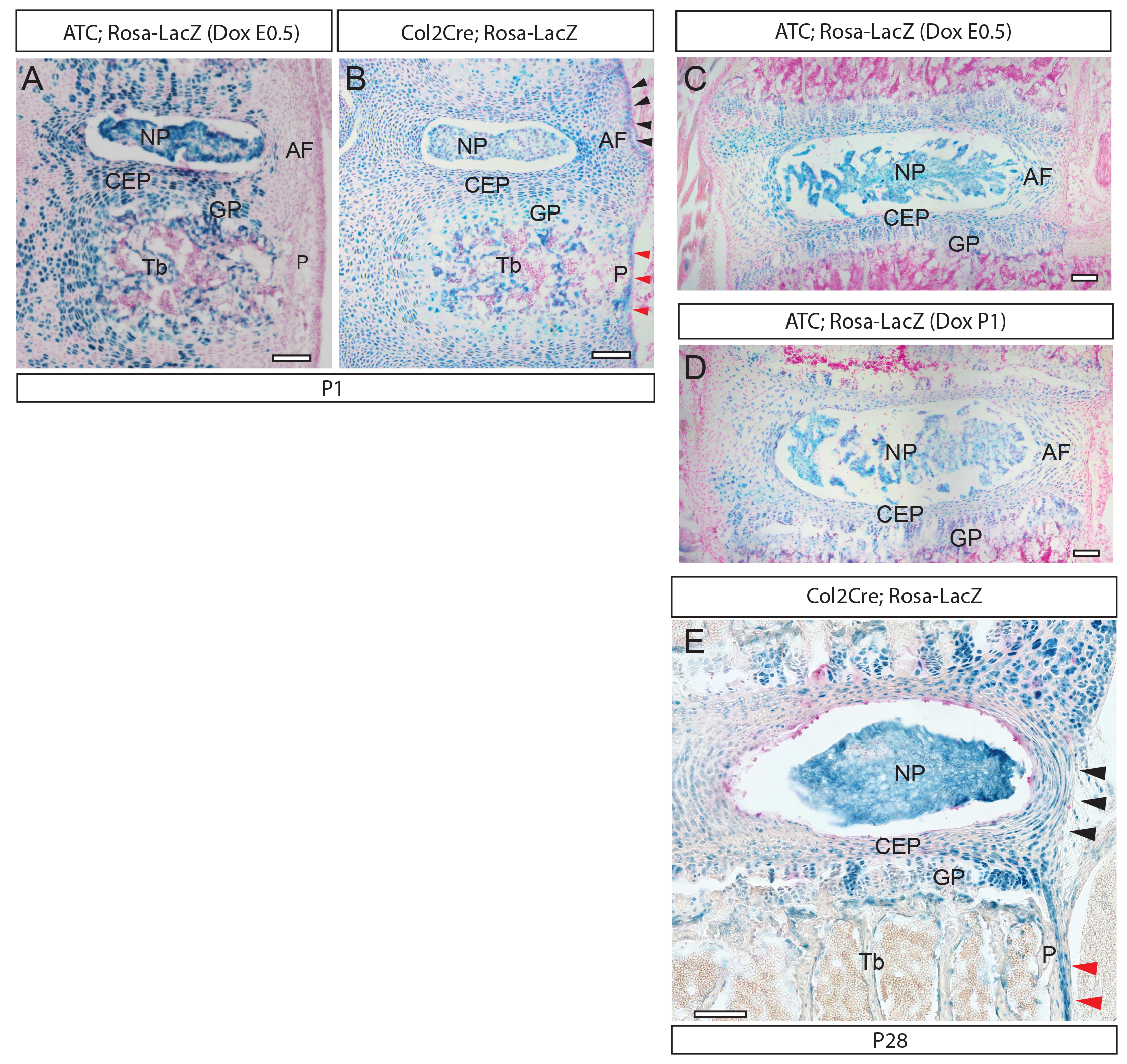

Supplement: S3 Fig — More robust recombination (blue signal) in CEP, GP, and AF of the IVD was observed in the Col2Cre; Rosa-LacZ mouse (B, E) compared with the ATC; Rosa-LacZ mouse when induced from E0.5-P20 (A, C) and P1-P20 (D). Recombination in periosteum (B, E, red arrows) and the outmost AF layers of the IVD (B, E, black arrows) was observed only in the Col2Cre; Rosa-LacZ mouse but not the ATC; Rosa-LacZ mouse. Scale bars: 100μm in (A-E). CEP- cartilaginous endplate, GP- growth plate, AF- annulus fibrosis, NP- nucleus pulposus, Tb- trabecular bone, and P- periosteum. (TIF) [file pgen.1008096.s003.tif]

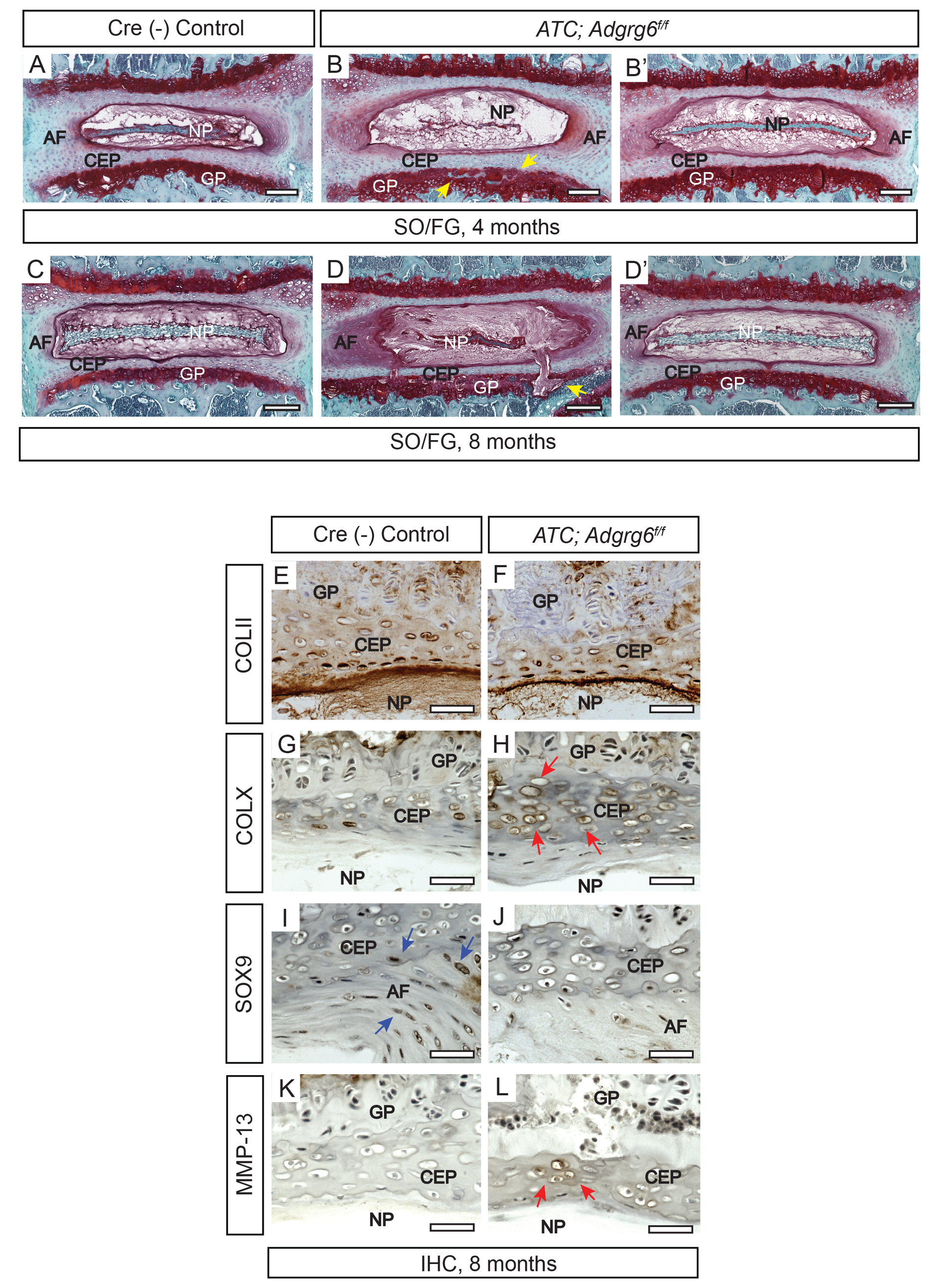

Supplement: S4 Fig — (A-D’) Representative 4-month-old (A-B’) or 8-month-old (C-D’) mouse IVDs stained with Safranin-O/Fast green (SO/FG). (Induced from P1-P20. For A-B’, n = 3 for controls and n = 5 for mutants; for C-D’, n = 4 for each group). Minor growth plate erosion is observed by the age of four months in mutant mice (yellow arrowheads, B), while more severe endplate-oriented disc herniations were observed by the age of 8 months (yellow arrowheads, D). (B’) is an earlier midline sections of the same mutant IVD as shown in B. (D’) is a midline section of an adjacent mutant IVD as shown in D, showing no overt histopathology. (E-L) IHC analysis of 8-month-old Cre (-) Control and ATC;Adgrg6f/f mutant mouse IVDs (induced from P1-P20). Several protein markers of IVD health and disease are affected in ATC;Adgrg6f/f mutant IVD including decreased expression of healthy disc markers COLII and SOX9 (G, blue arrows), and increased expression of the hypertrophic marker COLX (F, red arrows) and extracellular matrix modifying enzyme MMP-13 (J, red arrows). (n = 3 for each group.) Scale bars: 100μm in (A-D’); 50μm in (E-L). CEP: cartilaginous endplate, GP: growth plate, AF: annulus fibrosis, and NP: nucleus pulposus. (TIF) [file pgen.1008096.s004.tif]

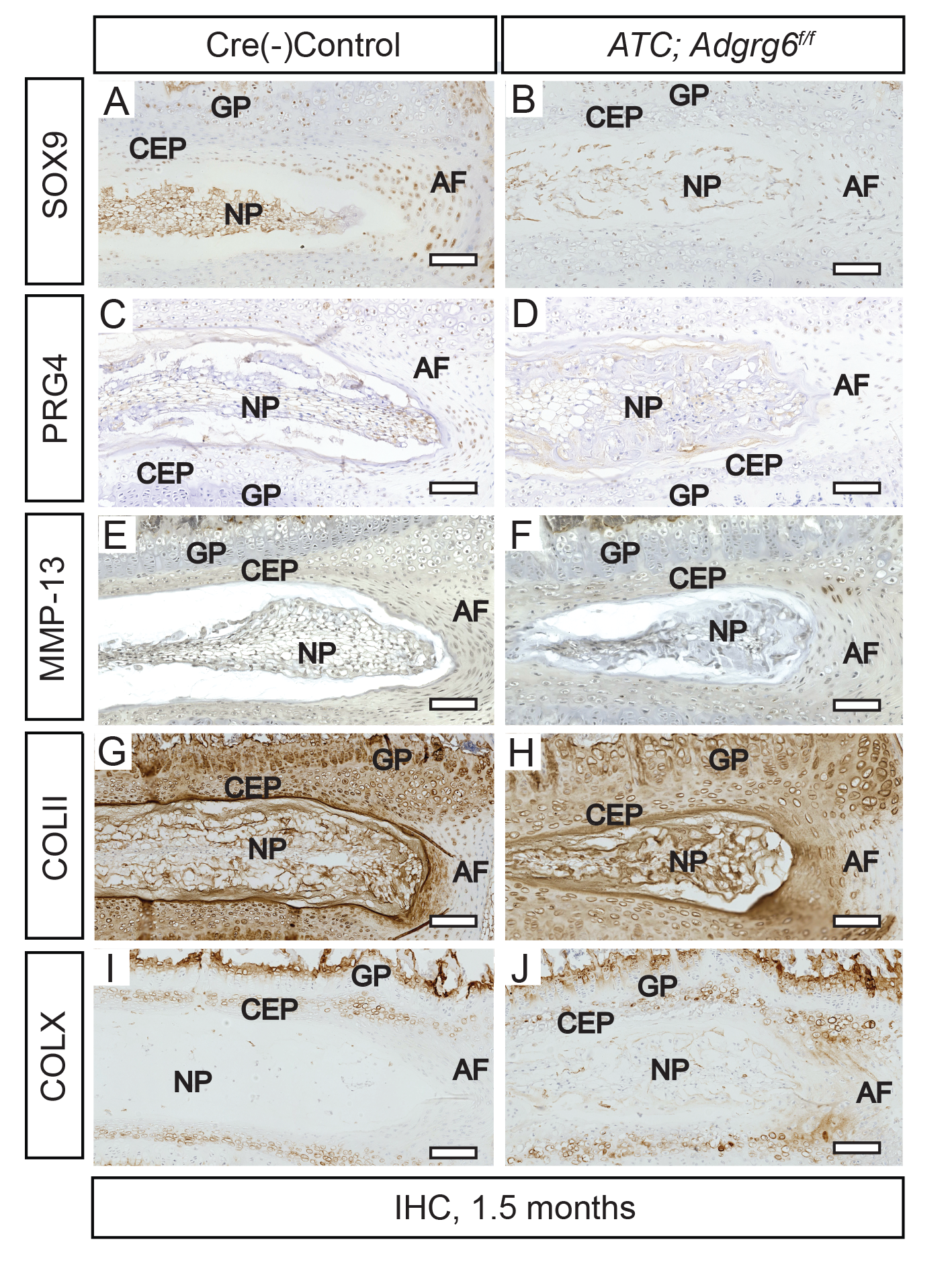

Supplement: S5 Fig — Large scale images of IHC analysis shown in Fig 2. IHC analysis of common markers of degenerative disc. ATC;Adgrg6f/f conditional mutant IVDs display reduced expression of markers of healthy disc: SOX9 (B), PRG4 (D), and COLII (H); and increased expression of extracellular matrix modifying enzymes MMP-13 (F), hypertrophic marker COLX (J). Scale bars: 100μm in (A-J). AF- annulus fibrosis, CEP- cartilaginous endplate, GP- growth plate, and NP- nucleus pulposus. (TIF) [file pgen.1008096.s005.tif]

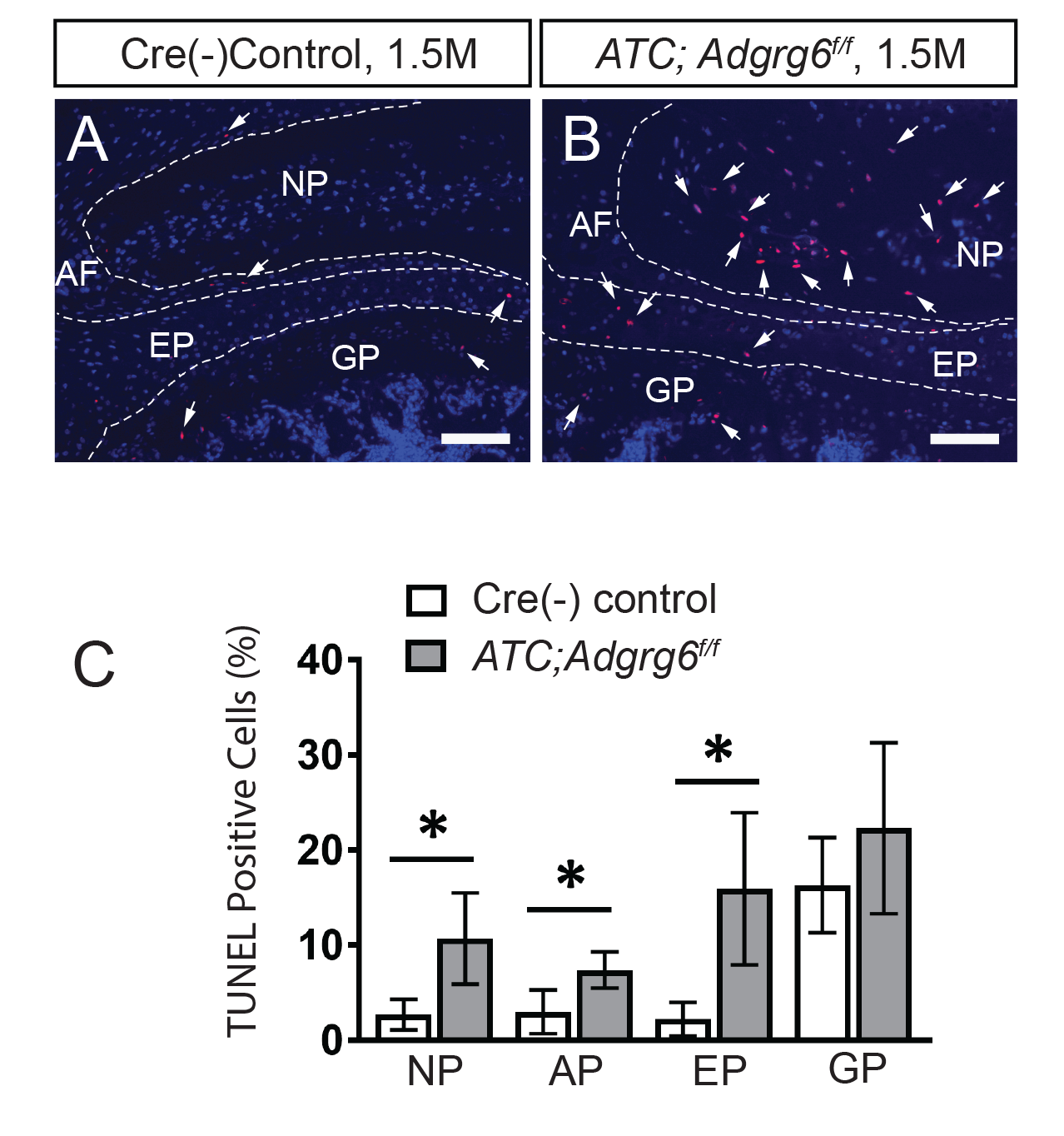

Supplement: S6 Fig — (A, B) TUNEL (red fluorescence) staining of 1.5-month-old ATC;Adgrg6f/f mutants (B, white arrows) display increased TUNEL positive cells compared to Cre (-) control (A) mice. (C) Graph of the ratio of TUNEL positive cells to total cells (DAPI) (n = 3 for each group, three to five IVDs were analyzed/mouse. Bars represent mean ± SD. *p≤0.05, two-tailed Student's t Test). AF- annulus fibrosis, CEP- cartilaginous endplate, GP- growth plate, and NP- nucleus pulposus. (TIF) [file pgen.1008096.s006.tif]

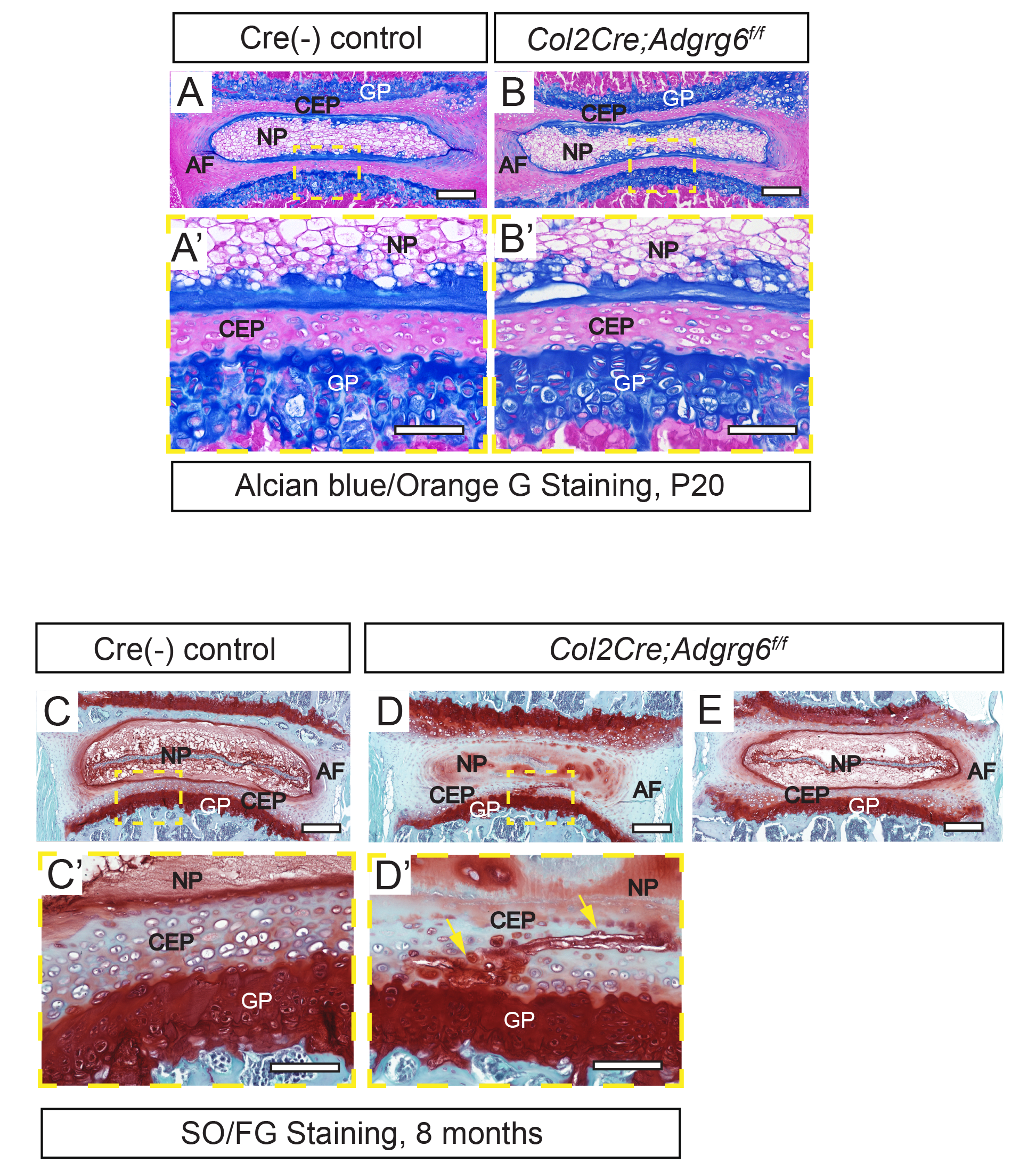

Supplement: S7 Fig — (A-B’) Representative medial-sectioned mouse IVDs stained with Alcian blue/Orange G of Cre (-) control (A and A') and Col2Cre;Adgrg6f/f mutant (B, B') mice at P20 (n = 3 for each group). No overt histopathology was observed in mutant mice at this young age. (C-E) Representative mouse IVDs stained with Safranin-O/Fast green (SO/FG) of Cre (-) control (C and C') and Col2Cre;Adgrg6f/f mutant (D, D', and E) mice by the age of 8 months (n = 3 for each group). Endplate-oriented herniations is indicated with yellow arrows. These herniations are very hard to be captured by histological analysis (D is out of the typical plane of section). E is an earlier midline section of the same mutant IVD as shown in D, showing no overt histopathology. Scale bars: 200μm in (A, B) and (C-E), and 50μm in (A’, B’) and (C’, D’). AF- annulus fibrosis, CEP- cartilaginous endplate, GP- growth plate, and NP- nucleus pulposus. (TIF) [file pgen.1008096.s007.tif]

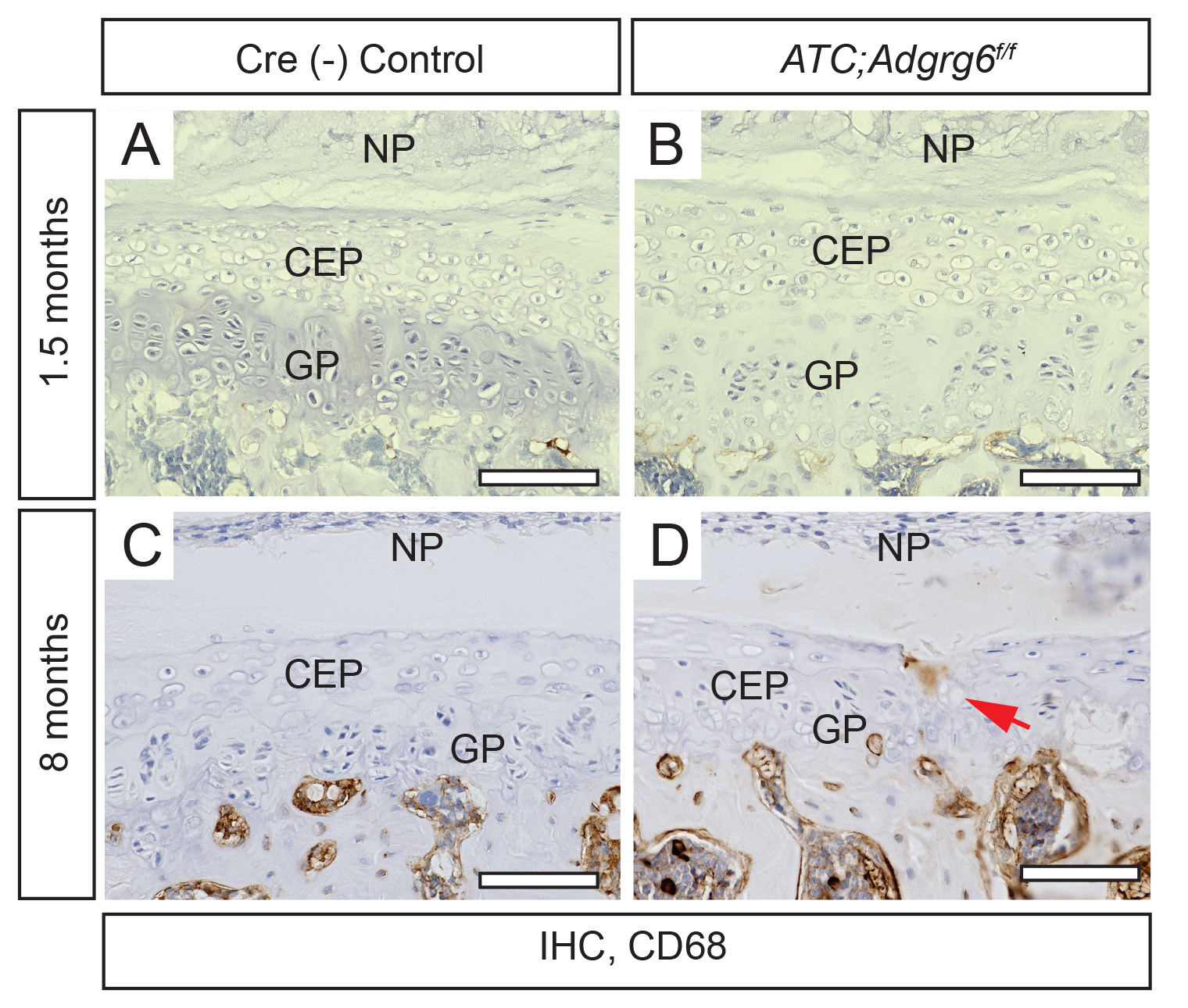

Supplement: S8 Fig — (A-D) IHC analysis of macrophage marker shows no strong signal of CD68 in ATC;Adgrg6f/f mutant mouse IVD at 1.5months (A, B), or 8 months of age (C, D), except for some background signal at the herniation site (red arrow, D). (Induced from E0.5-P20, n = 3 for each group.) Scale bars: 50μm in (A-D). CEP- cartilaginous endplate, GP- growth plate, and NP- nucleus pulposus. (TIF) [file pgen.1008096.s008.tif]

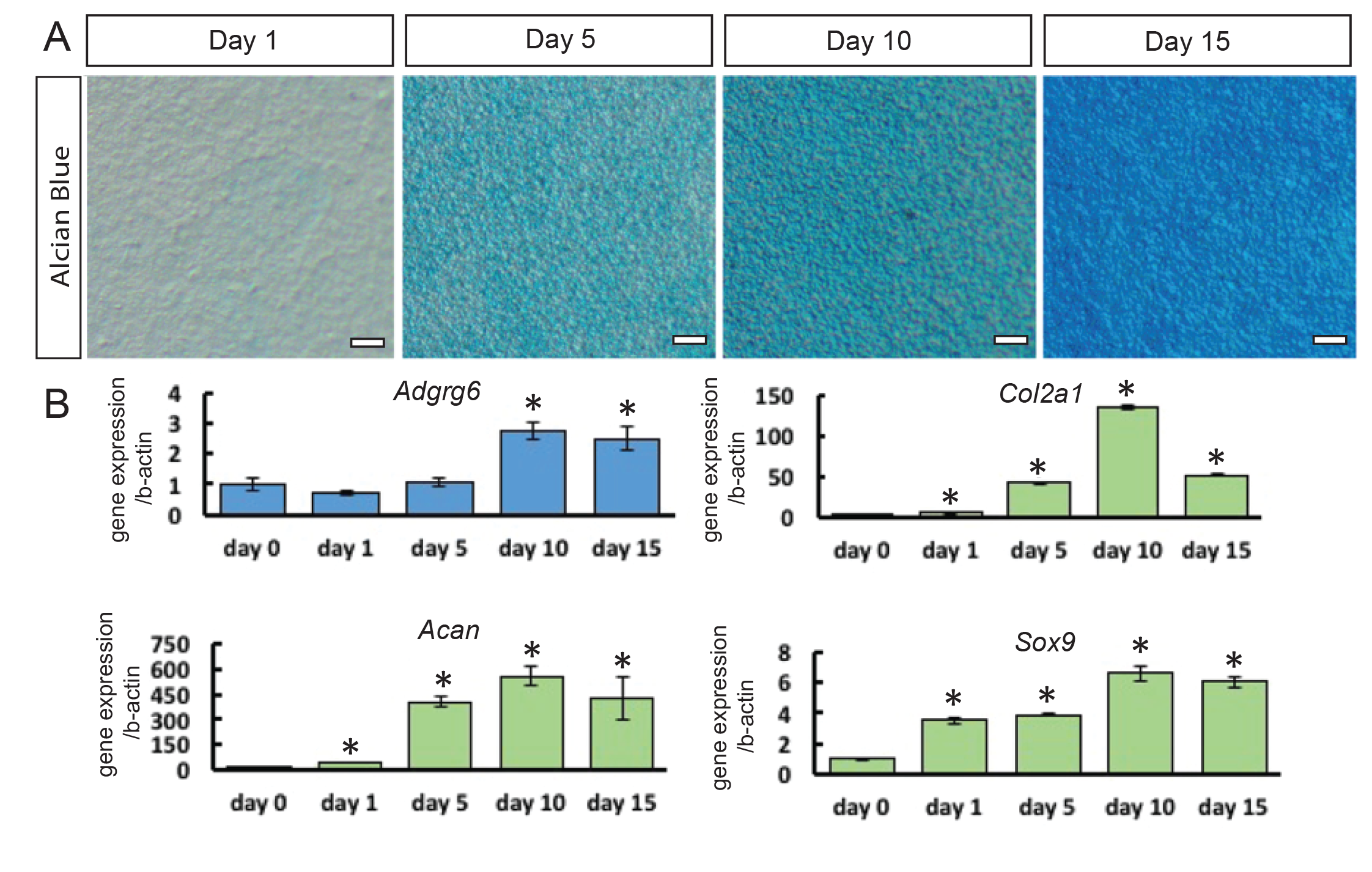

Supplement: S9 Fig — (A) Alcian blue staining on ATDC5 cell culture during the maturation process. (B) Expression profiles of Adgrg6, Col2a1, Acan, and Sox9 during ATDC5 cell maturation. The expression level of Adgrg6 was gradually increased alone with other chondrogenesis markers including Col2a1, Acan, and Sox9. (n = 3 biological replicates and representative result is shown. Bars represent mean ± SD. *p≤0.05, two-tailed Student's t Test). Scale bars: 100μm in A. (TIF) [file pgen.1008096.s009.tif]

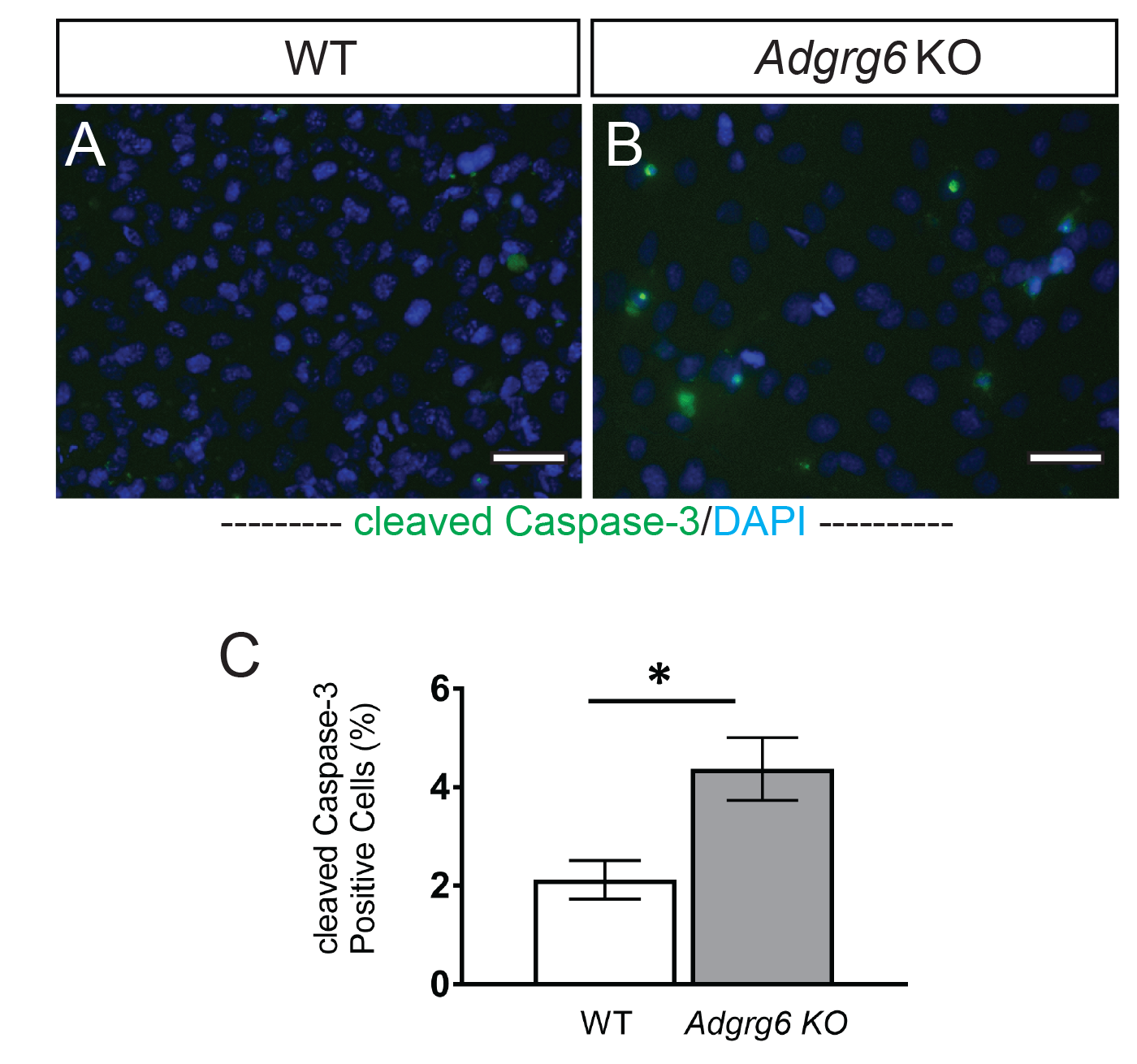

Supplement: S10 Fig — (A, B) Immunofluorescence against cleaved-Caspace-3 (green) and DAPI staining (blue) and (C) quantification showing increased apoptosis in Adgrg6 KO cells during maturation (10 days). (n = 3 biological replicates and representative result is shown. Bars represent mean ± SD. *p≤0.05, two-tailed Student's t Test). Scale bars: 50μm in (A, B). (TIF) [file pgen.1008096.s010.tif]

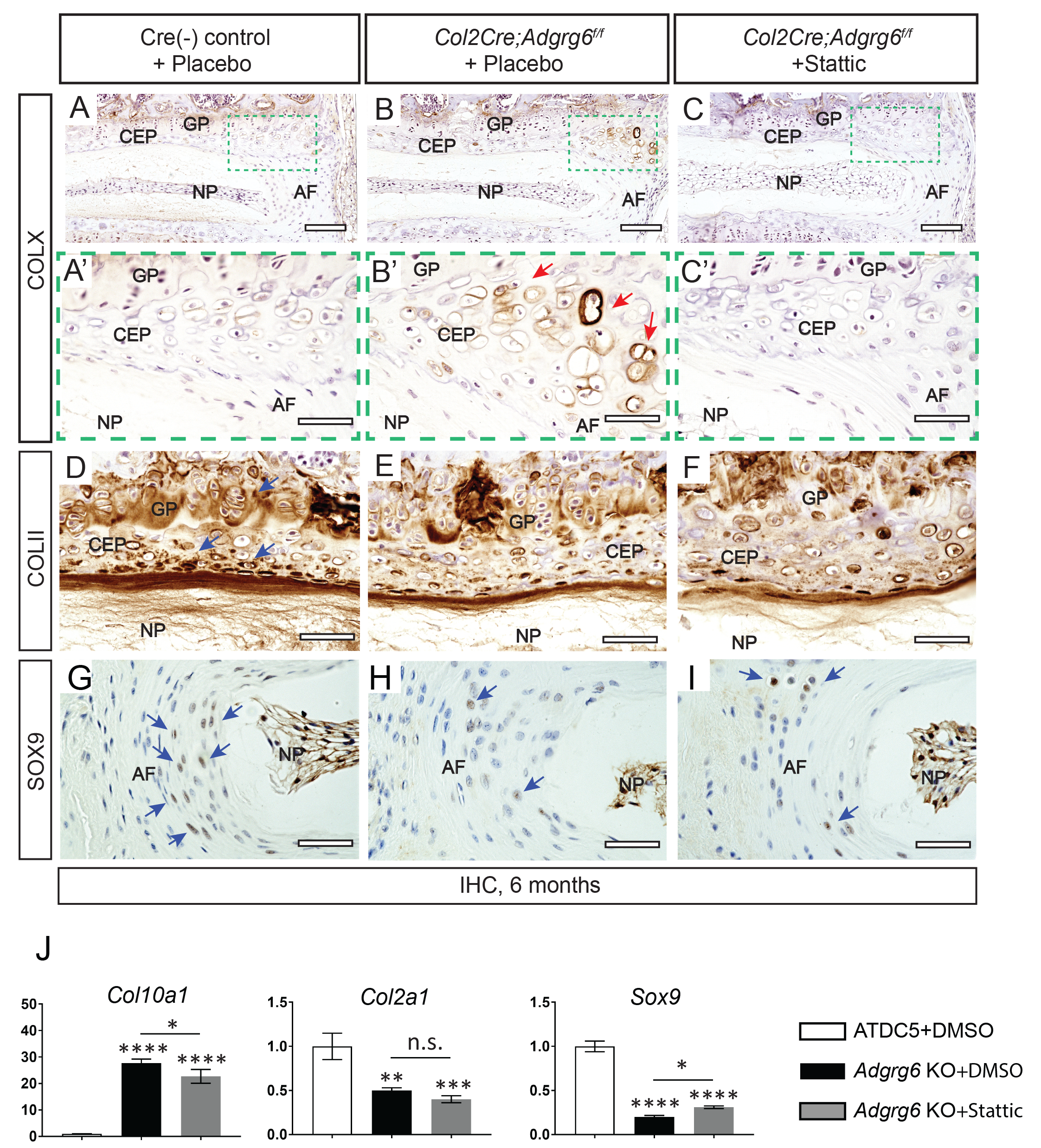

Supplement: S11 Fig — (A-C’) IHC analysis of COLX in 6-month-old Cre (-) control (A, A’) and Col2Cre;Adgrg6f/f mutant mice with (C, C’) or without (B, B’) Stattic treatment. Ectopic COLX expression was observed in CEP of the mutant mice (red arrows, B’), which is rescued after Stattic treatment (C’). (D-I) IHC analysis of COLII and SOX9 in 6-month-old Cre (-) control (A, D) and Col2Cre;Adgrg6f/f mutant mice with (F, I) or without (E, H) Stattic treatment. Reduced COLII and SOX9 expression was observed in Col2Cre;Adgrg6f/f conditional mutant mice compared with Cre (-) control (blue arrows, D, G), but no obvious improvement was observed after Stattic treatment (F, I). (n = 3 for each group). (J) qPCR analyses revealed that Col10a1 and Sox9 expression in Adgrg6 KO ATDC5 cells was partially rescued after Stattic treatment (10nM for 10 days), however the expression of Col2a1 was not significantly changed (n = 3 biological replicates and representative result is shown. Bars represent mean ± SD. *p≤0.05, **p≤0.01, ***p≤0.001, ****p≤0.0001, One way ANOVA followed by Tukey HSD test. n.s, not significant.) Scale bars: 100μm in (A-C), and 50μm in (A’-I). AF- annulus fibrosis, CEP- cartilaginous endplate, GP- growth plate, and NP- nucleus pulposus. (TIF) [file pgen.1008096.s011.tif]

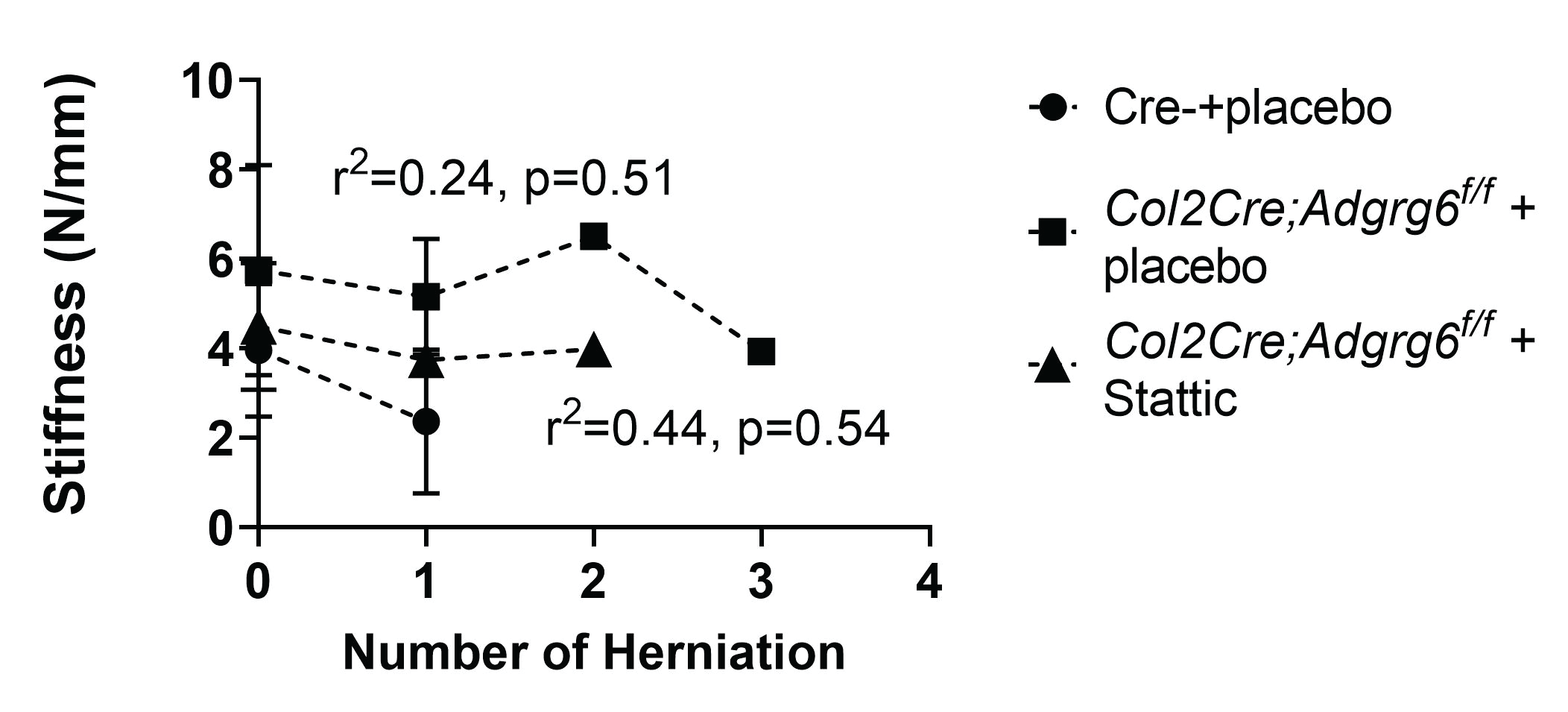

Supplement: S12 Fig — Correlation analysis (Pearson’s r) between number of herniation and disc stiffness were performed on 6-month-old mice from three experimental groups: four placebo-treated Cre (-) controls; three placebo-treated Col2Cre;Adgrgf/f mutants; and three Stattic-treated Col2Cre;Adgrgf/f mutants as shown in Fig 6. Dots plotted by mean ± SD. No correlation was detected. (TIF) [file pgen.1008096.s012.tif]
